# Long-Term Obesity and Biological Aging in Young Adults

**DOI:** 10.1001/jamanetworkopen.2025.20011

**Published:** 2025-07-11

**Authors:** Paulina Correa-Burrows, Raquel Burrows, Cecilia Albala, Carlos Sepúlveda, Felipe Salech, Rodrigo Troncoso, Daniel Bunout, Christian Gonzalez-Billault

**Affiliations:** 1 Institute of Nutrition & Food Technology, Universidad de Chile, Santiago, Chile; 2 Geroscience Center for Brain Health and Metabolism (GERO), Santiago, Chile; 3 Institute of Health Sciences, Universidad de O’Higgins, Rancagua, Chile; 4 Innovation Center, Clínica MEDS, Santiago, Chile; 5 Department of Neuroscience, Faculty of Medicine, Universidad de Chile, Santiago, Chile; 6 Advanced Center for Chronic Diseases (ACCDiS), Santiago, Chile; 7 Department of Biology, Faculty of Science, Universidad de Chile, Santiago, Chile; 8 The Buck Institute for Research on Aging, Novato, California

## Abstract

**Importance:**

It remains unclear whether obesity accelerates biological aging, potentially leading to early-onset chronic diseases.

**Objective:**

To investigate the association between long-term obesity and the expression of biochemical aging markers in younger adults.

**Design, Setting, and Participants:**

This multiple-events case-control study, conducted from April 5, 2022, to June 29, 2023, was embedded in the Santiago Longitudinal Study, a prospective Chilean birth cohort of adults aged 28 to 31 years among whom health and nutrition data were collected from September 1992 onward.

**Exposure:**

Body mass index (BMI) trajectory across the life course, recorded multiple times since birth. Group 1 had healthy BMI across the life course, group 2 had persistent obesity since adolescence, and group 3 had persistent obesity since childhood.

**Main Outcomes and Measures:**

Smoothed BMI trajectories (cubic polynomials) were used to estimate obesity duration. Primary outcomes were DNA methylation–based age and telomere length (TL). Secondary outcomes included levels of aging-related cytokines, growth factors, and adipomyokines.

**Results:**

In the sample of 205 adults (mean [SD] age, 28.9 [0.6] years; 100 females [49%]), 89 (43%) were in group 1, 43 (21%) in group 2, and 73 (36%) in group 3. Mean (SD) obesity duration was 12.9 (4.8) years in group 2 and 26.6 (2.3) years in group 3. Long-term obesity was associated with adulthood expression of biomarkers denoting antagonistic and integrative aging hallmarks, including mean (SD) hs-CRP (1.69 [2.1] vs 3.67 vs 4.24 [2.4] mg/L; *P* < .001; *f* = 0.57 [95% CI, 0.44-0.70]) and IL-6 (log, 0.69 [0.5] vs 1.03 [0.4] vs 0.99 [0.4]; *P* < .001; *f* = 0.53 [95% CI, 0.41-0.62]), as well as FGF-21, IGF-1, IGF-2, apelin, and irisin. Cohen *f* coefficient indicated a large effect size for the association of long-term obesity with adulthood expression of these markers.

**Conclusions and Relevance:**

In this multiple-events case-control study, long-term obesity was associated with the expression of biochemical aging markers in adults aged 28 to 31 years, consistent with epigenetic alterations, telomere attrition, chronic inflammation, impaired nutrient sensing, mitochondrial stress, and compromised intercellular communication. In young adults, chronic health issues may emerge from accelerated biological aging associated with long-term obesity.

## Key Points

**Question:**

Does obesity mimic the effects of aging in adults aged 28 to 31 years?

**Findings:**

In this case-control study of 205 participants from a Chilean prospective cohort, long-term obesity was associated with the expression of molecular aging signatures during young adulthood in females and males, including epigenetic modifications and telomere shortening. Exposure to long-term obesity was associated with epigenetic age exceeding chronological age by a mean of 15% to 16%, and in some cases, this difference reached 48%.

**Meaning:**

The findings suggest long-term obesity may trigger aging-related molecular signals in young adults.

## Teaser

This cohort study investigates whether long-term obesity in adults aged 28 to 31 years is associated with increased telomere attrition and epigenetic age exceeding chronological age.

## Introduction

Obesity is a major risk factor for the development of most noncommunicable chronic diseases. Extensive research shows that obesity reduces health span and life expectancy by increasing the risk of cardiometabolic, neoplastic, and musculoskeletal diseases,^1,2,3,4,5^ all conditions for which aging is the leading known risk factor. Typical consequences of aging, such as sarcopenia, atherosclerosis, insulin resistance, and declining adaptive immune function, are hastened by obesity. Furthermore, these health issues are increasingly seen in younger people^6,7,8,9,10^ and may indicate early signs of accelerated aging. Recently, 2 research groups reviewed the aging hallmarks and their potential link with obesity.^11,12^ Both concluded that the pathophysiological changes associated with obesity are similar to or contribute to those seen in aging, suggesting that obesity may accelerate the progressive decline in physiological integrity typically found in aging organisms. In 2023, additional hallmarks—chronic inflammation, dysbiosis, and altered macroautophagy—were identified, which are also prevalent in obesity, further supporting the hypothesis that obesity accelerates age-associated change, though it remains unproven.^13^

Obesity is associated with shortened lifespans and increased risk of early-onset chronic diseases.^14,15,16,17^ However, research is still lacking in understanding the specific molecular pathways and mechanisms connecting obesity and aging. Both share many physiological traits: systemic inflammation, telomere attrition, gut microbiome imbalance, mitochondrial dysfunction, impaired nutrient sensing, poor intercellular communication, altered proteostasis,^18,19,20,21,22,23,24^ cellular senescence,^25^ and age-related DNA hypomethylation.^26,27^ With an estimated 1 billion people expected to have obesity by 2030, we are approaching a future where the global population may be physiologically older than current sociodemographic data suggest, jeopardizing efforts for healthy, functional, and successful aging.^28^

A prospective cohort of males and females born in Chile in the 1990s could be an ideal model to test the hypothesis that obesity accelerates aging.^29^ At age 28 to 31 years, the mean body mass index (BMI, calculated as weight in kilograms divided by height in meters squared) in the cohort was 29, and 39% had obesity, with no differences by sex. Lipid profile, blood pressure, and pulse-wave velocity (PWV) suggested high cardiovascular risk. Prevalence of metabolic syndrome and metabolic dysfunction–associated steatotic liver disease (MASLD) increased from 15% to 24% at age 23 years to 38% to 55% at age 29 years, and 13.7% of participants already used glucose-, blood pressure–, or cholesterol-lowering medication as early as age 28 to 31 years. These clinical findings suggest that chronic exposure to obesity may have led these young adults to age faster than what is considered physiologically normal. We investigated whether biochemical aging signatures coexist with this dysfunctional cardiometabolic profile. Our hypothesis was that long-term obesity would be associated with these aging signatures in young adulthood.

## Methods

The institutional review board at the Institute of Nutrition & Food Technology, Universidad de Chile, approved this multiple-events case-control (MECC) study. Participants gave written informed consent. The Strengthening the Reporting of Observational Studies in Epidemiology (STROBE) guideline was followed.

### Study Design and Participants

We collected blood samples from participants (April 5, 2022, to June 29, 2023) in the Santiago Longitudinal Study (SLS) (N = 947), Chile’s oldest birth cohort. The SLS began September 1992 to study the effects of nutrition on children’s health, with follow-up assessments at ages 1, 5, 10, 12, 14, 16, 19, 21, 23, and 29 years. Enrollment criteria, rationale, and description of each assessment wave are described elsewhere.^9,29,30,31^ A flowchart for the cohort from its beginnings to the present day is in eFigure 1 in Supplement 1. A MECC design embedded in the SLS was conducted for this study. MECC involves a defined cohort from which participants are chosen for further measurements. MECC outperforms case-cohort and nested case-control designs by reducing bias and improving data analysis efficiency.^32^ We recruited females and males with complete data in all assessment waves who had maintained a healthy BMI across the life course (group 1), who had persistent obesity since adolescence (group 2), or who had persistent obesity since early childhood (group 3). Details on recruitment and selection criteria for this study were previously described.^31^ A sample size of 205 participants allowed comparing 3 groups, permitting up to 10 covariates at α = .05, 1 − β = 0.9, and *f* = 0.25. A comparison of included vs excluded SLS participants is in eTable 1 and eAppendix 1 in Supplement 1.

### Exposure

BMI trajectory across the life course, estimated from weight and height measured several times from birth to adulthood, was standardized with World Health Organization references.^33^ Reference values for females and males aged 19 years or older were used to standardize adulthood BMI. We used a cubic polynomial spline to interpolate each participant’s BMI trajectory across the life course. This method uses data points from original measurements and splines to smooth the transition between data points. Spline interpolation is preferred over other polynomial interpolation methods, because it can be used for segments and entire data series. It also allows for small interpolation errors.^34^ In addition, this method provides a smooth parametric curve when dealing with sparse data,^35^ particularly if the spline departs from the original data points, as was the case in this cohort. Following the method of Correa-Burrows et al,^30^ we obtained individual BMI trajectories from birth to adulthood. Then we estimated the timing of obesity onset and duration in participants with obesity. BMI trajectories across the life course are presented in eFigure 2 in Supplement 1, and a methodological note on trajectory modeling is given in eAppendix 2 in Supplement 1.

### Main Outcomes

The main outcomes were epigenetic age and telomere length. A 25-mL blood sample collected in EDTA tubes was taken from each participant during a morning assessment in our clinic. The same morning, peripheral blood mononuclear cells (PBMCs) were separated with Ficoll-Paque density gradient (GE HealthCare). DNA was extracted from PBMCs with DNeasy Blood & Tissue Kits (QIAGEN). One microgram of purified DNA was sent in batches of 96 to the Clock Foundation (Torrance, CA), preserving the cold chain. The Infinium MethylationEPIC BeadChip array (Illumina) was used to analyze over 850 000 CpG methylation sites in each sample across the genome, with samples applied randomly. Mean interarray correlation, which measures how similar (correlated) a given sample is compared with the other samples in the dataset, was 0.98 in our sample. DNA methylation data underwent thorough quality assessment using standard checks, including principal component analysis and visualizations through dendrograms and density plots. To reduce possible batch effect biases, the DNA methylation data were normalized.^36^ Aside from computing well-established first- and second-generation epigenetic clocks, the method also provides a methylation-based estimation of leukocyte telomere length (TL). For analysis, we used epigenetic age estimated with Horvath^36^ and GrimAge^37^ DNA methylation–based age clocks. We also computed the absolute and comparative differences between chronological age at assessment and methylation-based ages.

### Secondary Outcomes

The secondary outcomes were aging-related cytokines, growth factors, and myokines. Plasma levels of the proteins insulinlike growth factor 1 (IGF-1) and IGF-2; fibroblast growth factor 21 (FGF-21); growth differentiation factor 15 (GDF-15) and GDF-11; interleukin 2 (IL-2), IL-6, and IL-10; tumor necrosis factor α (TNF-α); and leptin, apelin, myostatin, osteonectin, irisin, oncostatin, and musclin were determined with the Luminex system (Luminex Corp). The Bio-Plex 200 platform (Bio-Rad Laboratories) was used with the following kits: MILLIPLEX MAP Human Cytokine-Chemokine Bead Panel (Merck Millipore) for IL-2, IL-6, IL-10, and TNF-α; MILLIPLEX Human Myokine Magnetic Bead Panel (Merck Millipore) for apelin, myostatin, irisin, musclin, musclin, osteonectin, and oncostatin; MILLIPLEX Human Aging Magnetic Bead Panel 1 (Merck Millipore) for GDF-11, GDF-15, FGF-21, and leptin; and MILLIPLEX MAP Human IGF-I, II Magnetic Bead Panel (Merck Millipore) for IGF-1 and IFG-2. Reagents were applied or prepared following manufacturer guidelines. Samples were measured in duplicate. For analysis, all variables were log-transformed. Serum high-sensitivity C-reactive protein (hs-CRP) level was measured with a sensitive latex-based immunoassay (eTable 2 in Supplement 1).

### Additional Measurements

Cardiometabolic profiling was also performed. Waist circumference (WC) was measured with a flexible tape at the midpoint between the last rib and the iliac crest. Systolic and diastolic blood pressures were measured 3 times after 15 minutes at rest in the upper arm using an oscillometric monitor; mean values were analyzed. Aortic PWV was measured using a Mobil-O-Graph (Cardiac Monitoring Service) oscillometer placed in the upper arm. After an 8- to 12-hour overnight fast, glucose, insulin, total cholesterol, triglycerides, and high-density lipoprotein (HDL) cholesterol levels were determined. Blood glucose level was measured with an enzymatic colorimetric test. Radioimmunoassay was used to determine insulin level. The dry analytical method was used to determine cholesterol profile (Ortho Clinical Diagnostics). Homeostatic model assessment (HOMA) quantified insulin resistance (HOMA-IR) and β-cell functioning (HOMA-β). Metabolic syndrome was diagnosed with the American Heart Association; National Heart, Lung, and Blood Institute; and International Diabetes Federation statement.^38^ A continuous metabolic syndrome severity score was computed,^39^ as well as a Hamaguchi liver score^40^ (range, 0-6, with higher scores indicating greater hepatic steatosis). Abdominal ultrasonography was performed to diagnose MASLD. Neck ultrasonography measured the carotid intima-media thickness. A description of the clinical and biochemical procedures, techniques, and references for diagnosis of cardiometabolic risk is in eTable 2 in Supplement 1.

### Statistical Analysis

We used Stata for Windows, version16.0 (StataCorp LLC), and XLSTAT-R, version 2024.3 (Addinsoft), for the data analyses. Data were expressed as mean (SD) or median (IQR), depending on the distribution’s normality. Cardiometabolic profile was compared using 1-way analysis of variance (ANOVA) with Tukey correction or the Kruskal-Wallis test with Dunn adjustment and repeated-measures ANOVA. To determine differences in aging-related markers based on BMI trajectories, we used analysis of covariance (ANCOVA) with Tukey correction, including sex as a covariate and the interaction of sex with BMI trajectory. Also, we calculated Cohen *f* as the effect size measure. Two-tailed paired *t* test was used to compare the same individual’s values of epigenetic age and chronological age; paired Hedges *g* measured the effect size.^41^ The Pettitt test assessed the consistency of the epigenetic age series across the entire sample, determining any potential change points within the series that could be associated with the BMI trajectory across the life course. Results were considered significant at 2-sided *P* < .05. A detailed description of the statistical methods used in this study is in eTable 2 in Supplement 1.

## Results

### Sample Description

The study involved 205 participants (mean [SD] age, 28.9 [0.6] years; 100 [49%] female and 105 [51%] male). In the sample, 43 participants (21%) had obesity since adolescence (group 2) and 73 (36%) since childhood (group 3); 89 (43%) always had a BMI in the healthy range (group 1). There was no association between sex and BMI trajectory. Participants’ chronological age ranged from 28.0 to 31.3 years, with no differences based on sex or BMI trajectory. Obesity onset in group 3 was at a mean (SD) age of 1.9 (0.7) years, whereas in group 2, it was at 15.8 (4.9) years. Mean (SD) obesity duration was 12.9 (4.8) years in group 2 and 26.6 (2.3) years in group 3. One participant (<1%) had type 2 diabetes, and 3 (1%) were taking metformin due to glucose intolerance. eTable 3 in Supplement 1 provides a sample description by sex.

Group 1 had lower WC, systolic blood pressure, PWV, insulin level, HOMA-IR, metabolic syndrome severity score, and Hamaguchi liver score (Table 1) than groups 2 and 3. No differences in these measures were found between groups 2 and 3. Group 1 participants maintained healthy cardiometabolic markers in adulthood, except for HDL cholesterol. Conversely, participants with long-term obesity showed elevated WC, systolic blood pressure, insulin level, HOMA-IR, and HOMA-β and reduced HDL cholesterol in adulthood. A median Hamaguchi score of 4 (IQR, 2-5) in group 2 and 4 (IQR, 3-6) in group 3 indicated a high likelihood of MASLD among these individuals. Mean fasting glycemia levels stayed within reference ranges for all groups; however, median HOMA-β values of 191.0% (IQR, 148.5%-271.8%) in group 2 and 191.7% (153.1%-313.4%) in group 3 suggested β-cell function nearly doubled compared with that of a healthy adult to keep glucose levels within physiological ranges. Metabolic syndrome severity and WC differed across all groups. The similarities in the cardiometabolic profile in participants from groups 2 and 3 suggested a similar degree of cardiometabolic dysfunction or damage in adulthood regardless of age at obesity onset. As expected, groups 2 and 3 displayed significantly higher prevalence of hyperglycemia, hyperinsulinemia, insulin resistance, inflammation, arterial stiffness, metabolic syndrome, and MASLD than group 1. Notably, no between-group differences were observed in the prevalence of low HDL cholesterol, and the frequency was markedly high even in group 1 (eTable 4 in Supplement 1). We observed that cardiometabolic markers changed as individuals moved from adolescence to adulthood. However, the health impact was greater for those with long-term obesity (eFigure 3 in Supplement 1).

**Table 1.** Between-Group Comparison of Participants’ Cardiometabolic Profile During the Assessment Wave at Age 29 Years

Variable	Group (N = 205)^a^			ANOVA for multiple comparisons^b^		Effect size Cohen *f* (95% CI)^d^
	1 (n = 89)	2 (n = 43)	3 (n = 73)	*P* value	Post hoc analysis^c^	
BMI	23.3 (2.1)	34.3 (4.4)	37.7 (5.9)	<.001	ABC	1.25 (1.05-1.35)
Waist circumference, cm						
Females	74.1 (6.8)	96.9 (9.6)	107.2 (13.5)	<.001	ABC	1.35 (1.12-1.47)
Males	83.7 (6.5)	106.2 (10.1)	112.3 (12.3)	.001	ABC	1.29 (1.09-1.41)
Blood pressure, mm Hg						
Systolic	115 (106 to 124)	122 (118 to 131)	125 (117 to 134)	.001^e^	ABB	0.05 (0.02-0.08)
Diastolic	75 (66 to 78)	77 (71 to 85)	76 (71 to 83)	.39^e^	NA	NA
Pulse-wave velocity, m/s	5.1 (0.3)	5.4 (0.3)	5.4 (0.3)	<.001	ABB	0.09 (0.04-0.17)
CIMT, mm						
Left	0.48 (0.06)	0.49 (0.07)	0.50 (0.07)	.29	NA	NA
Right	0.45 (0.06)	0.47 (0.05)	0.48 (0.07)	.14	NA	NA
Fasting blood glucose, mg/dL	91.1 (7.6)	92.1 (11.1)	97.8 (32.6)	.06	NA	NA
Fasting insulin, μUI/L	10.4 (7.6 to 12.7)	14.3 (10.2 to 19.2)	16.7 (11.2 to 23.7)	<.001^e^	ABB	0.14 (0.07-0.22)
HOMA						
Insulin resistance ratio	2.1 (1.5 to 2.7)	3.2 (2.4 to 4.6)	3.9 (2.6 to 6.1)	<.001^e^	ABB	0.12 (0.05-0.19)
β, %	133.4 (95.3 to 192.0)	191.0 (148.5 to 271.8)	191.7 (153.1 to 313.4)	<.001^e^	ABB	0.11 (0.05-0.16)
Fasting triglycerides, mg/dL	84.1 (55.4 to 109.1)	115.6 (75.5 to 154.3)	121.3 (82.1 to 180.0)	.02^e^	ABB	0.02 (0.01-0.04)
Cholesterol, mg/dL						
HDL						
Females	25.9 (21.2 to 33.3)	23.8 (18.6 to 28.5)	21.3 (17.5 to 33.4)	.67^e^	NA	NA
Males	20.0 (18.2 to 25.0)	18.8 (16.5 to 26.2)	18.8 (14.1 to 22.6)	.68^e^	NA	NA
Total cholesterol	167.4 (144.1 to 197.3)	178.9 (154.0-205.1)	173.9 (137.9-197.7)	.31^e^	NA	NA
Metabolic syndrome severity, *z*-score SD	0.12 (−0.33 to 0.82)	0.72 (0.18 to 1.10)	1.00 (0.42 to 1.70)	<.001^e^	ABC	0.13 (0.05-0.21)
Hamaguchi liver score^f^	2 (1 to 3)	4 (2 to 5)	4 (3 to 6)	<.001^e^	ABB	0.14 (0.03-0.19)

Abbreviations: ANOVA, analysis of variance; BMI, body mass index, calculated as weight in kilograms divided by height in meters squared; CIMT, carotid intima-media thickness; HDL, high-density lipoprotein; HOMA, homeostatic model assessment; NA, not applicable.

SI conversion factors: To convert glucose to mmol/L, multiply by 0.0555; HDL to mmol/L, multiply by 0.0259; insulin to pmol/L, multiply by 6.945; total cholesterol to mmol/L, multiply by 0.0259; triglycerides to mmol/L, multiply by 0.0113.

^a^ Data are presented as mean (SD) or median (IQR). Group 1 participants always had a BMI in the healthy range, group 2 had obesity starting in adolescence and remaining into adulthood, and group 3 had obesity in early childhood and remaining into adulthood.

^b^ ANOVA with Tukey post hoc adjustment, except where otherwise indicated.

^c^ A indicates group 1; B, group 2; and C, group 3. ABB indicates that group 1 had mean values significantly different from those of groups 2 and 3 in the post hoc analysis for between-group differences, while the mean values in groups 2 and 3 did not significantly differ, and ABC indicates a significant difference between all 3 groups.

^d^ The effect size for the Kruskal-Wallis test was computed as the ε^2^ based on the H statistic: ε^2^ [*H*] = (*H* − *k* + 1)/(*n* − *k*), where *H* is the value obtained in the Kruskal-Wallis test, *k* is the number of groups, and *n* is the total number of observations. The ε^2^ estimate assumes values from 0 to 1; multiplied by 100, it indicates the percentage of variance in the dependent variable explained by the independent variable. The interpretation values commonly found in published literature are 0.01 to less than 0.06 (small effect), 0.06 to less than 0.14 (moderate effect), and 0.14 or greater (large effect).^19,42^ For the ANOVA test (Cohen *f* coefficient), 0.10 was a small effect size; 0.25, moderate; and 0.40, large.

^e^ From Kruskal-Wallis H tests with Dunn post hoc adjustment.

^f^ Hamaguchi liver score ranges from 0 to 6, with higher scores indicating greater hepatic steatosis.

### Main Outcomes

DNA methylation–based age from Horvath (Figure, A) and GrimAge (Figure, B) clocks of participants in groups 2 and 3 consistently exceeded their chronological age at the time of assessment, while in group 1, DNA methylation–based age from Horvath and GrimAge clocks tended to be approximately equivalent to chronological age. The Pettitt test confirmed the epigenetic age series were nonuniform. ANCOVA examined between-group differences for epigenetic-based aging biomarkers. Group 1 had lower mean (SD) DNA methylation–based age (Horvath, 28.5 [2.5] years; GrimAge, 26.3 [3.5] years) and greater TL (8.01 [0.36] kb) than group 2 (Horvath, 34.1 [3.8] years; GrimAge, 31.6 [3.7] years; TL, 7.46 [0.32] kb) and group 3 (Horvath, 34.5 [4.6] years; GrimAge, 32.5 [3.9] years; TL, 7.42 [0.26] kb) (all *P* < .001). The Cohen *f* values for these markers (Horvath clock age: 0.71 [95% CI, 0.58-0.84]; GrimAge clock age: 0.65 [95% CI, 0.52-0.78]; TL: 0.81 [95% CI, 0.68-0.95]) indicate that there was a large effect size for the association of BMI trajectory with the epigenetic-based aging profile in adulthood (Table 2). The pairwise comparison found no significant differences in DNA methylation–based age from the Horvath clock compared with chronological age for group 1, but Horvath DNA methylation–based age was significantly higher than chronological age in both group 2 (by a mean [SD] of 4.4 [3.7] years, or 15.2% [13.2%]) and group 3 (by 4.7 [4.2] years, or 16.4% [14.1%]) (both *P* < .001). For some participants, the difference was as much as 48%. Hedges *g* values indicated a large difference between Horvath clock DNA methylation–based age and chronological age in group 2 (−1.60; 95% CI, −2.08 to −1.11) and group 3 (−1.51; 95% CI, −1.87 to −1.14), with 38 participants (87.3%) in group 2 and 63 (85.9%) in group 3 having DNA methylation–based age higher than the group’s mean chronological age. Additionally, the chance that a randomly selected person would have a Horvath clock DNA methylation–based age higher than their chronological age was 87.3% in group 2 and 85.9% in group 3 (Table 3). A similar pattern was observed when comparing DNA methylation–based age from the GrimAge clock with chronological age.

**Figure.**
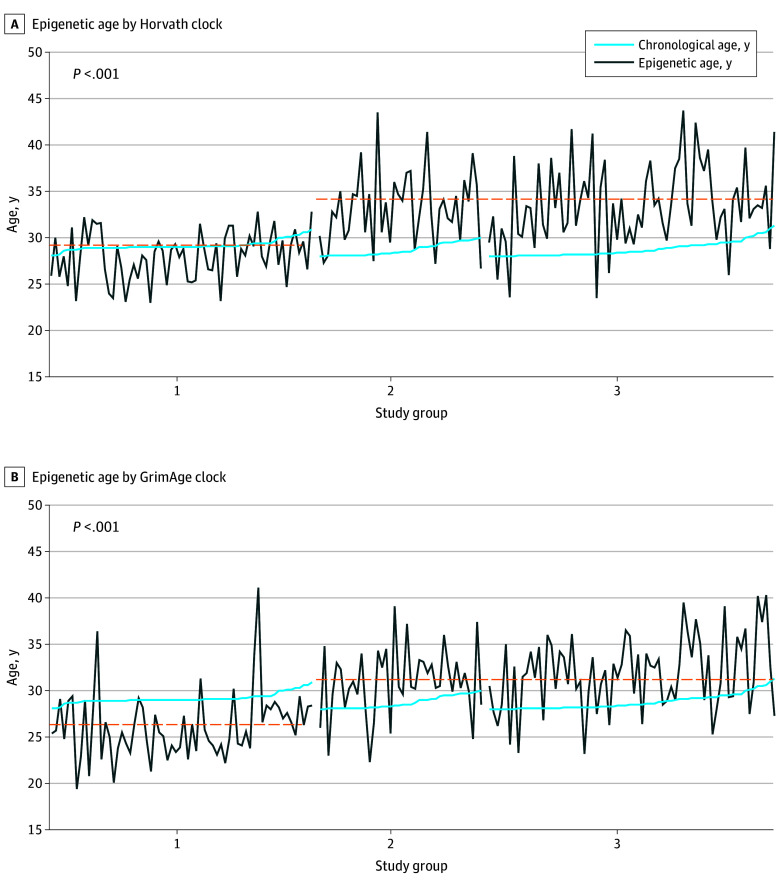
Data Visualization of Chronological vs Epigenetic Age in the Sample Group 1 participants always had a body mass index in the healthy range, group 2 had obesity starting in adolescence and remaining into adulthood, and group 3 had obesity in early childhood and remaining into adulthood. A 2-tailed Pettitt test for homogeneity was used to detect change points in the data series. A, Pettitt test N = 5070. B, Pettitt test N = 5310. Horizontal dashed lines indicate the mean epigenetic age value for each of the 2 series identified by the Pettitt test.

**Table 2.** Epigenetic Age–Related Phenotype of Participants by BMI Trajectory

	Group (N = 205)^a^			Between-group difference^b^	Cohen *f* (95% CI)^c^
	1 (n = 89)	2 (n = 43)	3 (n = 73)		
Chronological age, y	28.9 (0.8)	28.7 (0.6)	28.8 (0.8)	NS	NA
LTL, kb	8.01 (0.36)	7.46 (0.32)	7.42 (0.26)	ABB	0.81 (0.68-0.95)
Horvath clock					
Age, y	28.5 (2.5)	34.1 (3.8)	34.5 (4.6)	ABB	0.71 (0.58-0.84)
Acceleration, y	−0.4 (2.5)	4.4 (3.7)	4.7 (4.2)	ABB	0.77 (0.64-0.90)
Acceleration, %	−1.4 (8.6)	15.2 (13.2)	16.4 (14.1)	ABB	0.77 (0.63-0.91)
GrimAge clock					
Age, y	26.3 (3.5)	31.6 (3.7)	32.5 (3.9)	ABB	0.65 (0.52-0.78)
Acceleration, y	−2.8 (3.5)	2.2 (3.6)	3.1 (3.8)	ABB	0.71 (0.58-0.85)
Acceleration, %	−10.2 (1.2)	7.7 (1.3)	10.7 (1.3)	ABB	0.71 (0.58-0.85)

Abbreviations: BMI, body mass index; LTL, leukocyte telomere length; NA, not applicable; NS, not significant.

^a^ Values for the main outcome, expressed as mean (SD). Models were adjusted for sex and the interaction of sex with BMI trajectory across the life course. Group 1 participants always had a BMI in the healthy range, group 2 had obesity starting in adolescence and remaining into adulthood, and group 3 had obesity in early childhood and remaining into adulthood.

^b^ Analysis of covariance with Tukey adjustment (Tukey post hoc analysis for between-group differences). A indicates group 1; and B, group 2; and C, group 3. ABB indicates that group 1 had values significantly different from those of groups 2 and 3, while the mean values in groups 2 and 3 did not differ.

^c^ The effect size for the difference was computed as the Cohen *f* coefficient. A small effect size was Cohen *f* of 0.10; moderate, 0.25; and large, 0.40.

**Table 3.** Pairwise Comparison of Chronological Age vs Epigenetic Age in Participants With Long-Term Obesity

Participants^a^	Age, mean (SD), y			Paired *t* test	*P* value	Paired Hedges *g*, mean (95% CI)	Cohen *d* (95% CI)	Cohen U3 index, %	Probability of superiority, %
	Chronological	Epigenetic	Difference						
**Horvath clock**									
Group 2	28.8 (0.7)	33.1 (3.7)	−4.46 (0.61)	−7.53	<.001	−1.60 (−2.08 to −1.11)	−1.61 (−2.10 to −1.12)	94.6	87.3
Group 3	28.7 (0.8)	33.5 (4.3)	−4.68 (0.51)	−9.57	<.001	−1.51 (−1.87 to −1.14)	−1.52 (−1.88 to −1.15)	93.6	85.9
**GrimAge clock**									
Group 2	28.7 (0.7)	30.9 (3.6)	−2.23 (0.58)	−3.96	<.001	−0.82 (−1.25 to −0.38)	−0.83 (−1.26 to −0.39)	79.4	71.9
Group 3	28.8 (0.8)	31.8 (4.0)	−3.00 (0.45)	−6.61	<.001	−1.03 (−1.37 to −0.68)	−1.03 (−1.38 to −0.68)	84.8	76.7

^a^ Group 2 participants had obesity starting in adolescence and remaining into adulthood, and group 3 participants had obesity in early childhood and remaining into adulthood.

### Secondary Outcomes

Considering the observed acceleration in the epigenetic clocks, several markers associated with the hallmarks of aging were analyzed. Evidence of inflammaging was found; as compared with groups 2 and 3, group 1 showed significantly lower mean (SD) levels of hs-CRP (1.69 [2.1] vs 3.67 [2.8] vs 4.24 [2.4] mg/L for groups 1, 2, and 3, respectively; *P* < .001; *f* = 0.57 [95% CI, 0.44-0.70]) and IL-6 (log, 0.69 [0.5] vs 1.03 [0.4] vs 0.99 [0.4]; *P* < .001; *f* = 0.53 [95% CI, 0.41-0.62]), as well as IL-2 and IL-10, with no differences between group 2 and group 3 (Table 4). Additionally, group 3 had elevated GDF-15 levels, a marker linked to cellular stress, compared with groups 1 and 2. However, no group differences were observed for GDF-11 or TNF-α. BMI trajectory was associated with growth factor signaling, with groups 2 and 3 having higher FGF-21 (log, 2.21 [0.2] vs 2.42 [0.2] vs 2.45 [0.2] for groups 1, 2, and 3, respectively; *P* < .001; *f* = 0.48 [95% CI, 0.36-0.60]) and leptin levels than group 1. IGF-1 levels were reduced in groups 2 and 3 compared with group 1 (log, 4.65 [0.2] vs 4.55 [0.2] vs 4.45 [0.3] for groups 1, 2, and 3, respectively; *P* < .001; *f* = 0.56 [95% CI, 0.44-0.69]), while IGF-2 levels were higher in group 1 (log, 5.54 [0.1] vs 5.46 [0.1] vs 5.44 [0.2]; *P* < .001; *f* = 0.49 [95% CI, 0.46-0.61]). Regarding intercellular communication impairment, group 1 had significantly lower levels of apelin (log, 5.49 [0.2] vs 5.65 [0.3] vs 5.63 [0.3] for groups 1, 2, and 3, respectively; *P* < .001; *f* = 0.42 [95% CI, 0.30-0.54]) and irisin (log, 7.71 [0.1] vs 7.83 [0.2] vs 7.80 [0.2]; *P* < .001; *f* = 0.46 [95% CI, 0.19-0.44]), as well as oncostatin, myostatin, and musclin, than groups 2 and 3, indicating disrupted signaling pathways in the latter groups. Conversely, group 1 had higher musclin levels, a myokine associated with muscle function, than groups 2 and 3.

**Table 4.** Aging-Related Cytokines, Adipokines, Myokines, and Growth Factors in Participants by BMI Trajectory

Biomarker	Group (N = 205)^a^			Between-group differences^b^	Cohen *f* (95% CI)^c^
	1 (n = 89)	2 (n = 43)	3 (n = 73)		
hs-CRP, mg/L	1.69 (2.1)	3.67 (2.8)	4.24 (2.4)	ABB	0.57 (0.44-0.70)
IL-2 (log)	0.52 (0.3)	0.60 (0.2)	0.63 (0.2)	ABB	0.25 (0.13-0.38)
IL-6 (log)	0.69 (0.5)	1.03 (0.4)	0.99 (0.4)	ABB	0.53 (0.41-0.62)
IL-10 (log)	2.18 (0.3)	2.27 (0.4)	2.36 (0.5)	ABB	0.28 (0.15-0.40)
TNF-α (log)	2.49 (0.5)	2.52 (0.5)	2.63 (0.6)	NS	NA
FGF-21 (log)	2.21 (0.2)	2.42 (0.2)	2.45 (0.2)	ABB	0.48 (0.36-0.60)
GDF-11 (log)	1.22 (0.05)	1.23 (0.05)	1.23 (0.05)	NS	NA
GDF-15 (log)	3.44 (0.1)	3.45 (0.2)	3.51 (0.1)	AAC	0.37 (0.24-0.49)
IGF-1 (log)	4.65 (0.2)	4.55 (0.2)	4.45 (0.3)	ABC	0.56 (0.44-0.69)
IGF-2 (log)	5.54 (0.1)	5.46 (0.1)	5.44 (0.2)	ABB	0.49 (0.46-0.61)
Leptin (log)^a^					
Females	4.31 (0.3)	4.63 (0.3)	4.71 (0.3)	ABB	0.45 (0.34-0.57)
Males	3.93 (0.4)	4.22 (0.2)	4.36 (0.2)	ABB	0.53 (0.41-0.65)
Apelin (log)	5.49 (0.2)	5.65 (0.3)	5.63 (0.3)	ABB	0.42 (0.30-0.54)
Myostatin (log)	7.06 (0.2)	7.19 (0.2)	7.16 (0.3)	ABB	0.28 (0.14-0.39)
Irisin (log)	7.71 (0.1)	7.83 (0.2)	7.80 (0.2)	ABB	0.46 (0.34-0.59)
Oncostatin (log)	1.59 (0.2)	1.72 (0.3)	1.70 (0.2)	ABB	0.38 (0.26-0.51)
Musclin (log)	6.21 (0.3)	6.08 (0.4)	6.04 (0.4)	ABB	0.47 (0.35-0.60)
Osteonectin (log)	5.18 (0.3)	5.27 (0.4)	5.34 (0.3)	ABB	0.31 (0.19-0.44)

Abbreviations: BMI, body mass index; hs-CRP, high-sensitivity C-reactive protein; FGF, fibroblast growth factor; GDF, growth differentiation factor; IGF, insulinlike growth factor; IL, interleukin; NA, not applicable; NS, no significant difference; TNF, tumor necrosis factor.

^a^ Values are expressed as mean (SD). To reduce skewness and for analysis, all variables except hs-CRP were log-transformed using natural logarithms; hence, variables are expressed in logarithmic units. Group 1 participants always had a BMI in the healthy range, group 2 had obesity starting in adolescence and remaining into adulthood, and group 3 had obesity in early childhood and remaining into adulthood.

^b^ Analysis of covariance with Tukey adjustment (Tukey post hoc analysis for between-group differences). A indicates group 1; B, group 2; and C, group 3. ABB indicates that group 1 had values significantly different from those of groups 2 and 3, while the mean values in groups 2 and 3 did not differ, and ABC indicates a significant difference between all 3 groups. Models were adjusted for sex and the interaction of sex with body mass index trajectory across the life course. Because sex was statistically significant in the analysis of covariance model, leptin models were rerun separately for males and females.

^c^ The effect size for the difference was computed as the Cohen *f* coefficient; 0.10 was a small effect size, 0.25 was moderate, and 0.40 was large.

## Discussion

We conducted comprehensive clinical, physiological, and biochemical evaluations on individuals aged 28 to 31 years from Chile’s oldest birth cohort to detect early expressions of molecular aging biomarkers and establish possible associations with long-term obesity. Also, we aimed to evaluate the association of this early aging phenotype with obesity-related cardiometabolic dysfunction. Findings suggest long-term obesity was associated with premature physiological decline, inducing molecular aging signatures as early as age 28 to 31 years. Signs included a 15.2% to 16.4% increase in epigenetic age compared with chronological age (with some individuals showing up to 48% increase), telomere attrition, chronic inflammation, impaired nutrient sensing, mitochondrial stress, and compromised intercellular communication.

The link between increased BMI and epigenetic aging was first reported by Horvath et al,^26^ who found hepatocytes aged 2.7 years for a 10-point BMI increase. A meta-analysis indicated BMI was associated with accelerated epigenetic aging across various clocks, with some studies identifying BMI as the greatest contributor.^43^ Our findings support this evidence and, for the first time to our knowledge, reveal the presence and extent of these connections in a Hispanic population, an underrepresented group in aging research; our findings also provide supporting evidence of a connection between obesity and epigenetic aging by evaluating biomarkers linked to the hallmarks of aging. All epigenetic-aging biomarkers were associated with long-term obesity. Notably, epigenetic changes and telomere attrition are primary aging hallmarks, believed to be the root causes of cell and tissue damage.^44^ Although our findings do not conclusively indicate which biochemical aging signatures appeared first, they suggest obesity has a greater impact on primary aging hallmarks than antagonistic and integrative hallmarks. Therefore, we have initial evidence that obesity may be associated with aging by affecting the molecular responses that initiate damage. How obesity might affect epigenetic regulation through endocrine, metabolic, and cellular senescence pathways is discussed in eAppendix 3 in Supplement 1.

Long-term obesity was associated with hs-CRP, IL-6, and leptin levels, all well-known biomarkers of systemic inflammation, a newly recognized aging hallmark stemming from epigenetic dysregulation, impaired autophagy, or buildup of senescent cells.^18^ Inflammation, in turn, favors other aging signs, such as poor intercellular communication.^13,18^ Participants with long-term obesity also presented dysregulated adipomyokines, a group of proteins at the muscle-organ crosstalk to the brain, adipose tissue, bone, liver, gut, pancreas, and vascular bed.^45,46^ Obesity and aging are both associated with dysregulated myokine secretion and signaling.^47,48^ Hence, it is unsurprising these proteins were elevated in participants with long-term obesity compared with control individuals with healthy weight—particularly apelin and irisin, which tend to decline with age. In still-young individuals, increasing these myokines may be a compensatory response to improve insulin sensitivity in obesity or the consequence of reduced sensitivity to its effects, as seen with insulin and leptin in obesity.^49,50^ Upregulated IL-6 and GDF-15 have also been used to feature cell senescence in vitro, particularly in the senescence-associated secretory phenotype.^51^

Upregulated insulin and downregulated IGF-1 and IGF-2 levels may denote impaired nutrient sensing in young adults. An elevated IGF-1 level relates to lowered disease risk in younger individuals, while higher levels in older adults relate to increased morbimortality.^52^ In individuals with obesity, a lower IGF-1 level may indicate higher disease risk. As IGF-1 tends to decrease with age, higher levels in young adults may be a youth marker.^52,53^ Reduced IGF-2 has been associated with aging in various organs and primordial germ cells while compromising mitochondrial functionality.^54,55^ In addition, upregulated FGF-21 and GDF-15 have been regarded as markers of mitochondrial stress and possibly dysfunction.^56^ Additional discussions on cytokines related to obesity, along with profiles of adipokines, myokines, and growth factors as signatures of molecular aging, can be found in eAppendix 4 and eTable 5 in Supplement 1.

Aging signatures were expressed similarly in participants with obesity (regardless of age at obesity onset), supporting that persistency rather than onset time is the key factor in obesity-related dysfunctions.^30,57,58^ Whether uniformity persists as these young adults approach middle or old age remains to be investigated. However, progression to disease is ongoing, as dysfunctional cardiometabolic biomarkers in individuals with long-term obesity mostly fell outside healthy ranges. Further discussion of the clinical implications of our findings is in eAppendix 5 in Supplement 1. The preeminence of cardiometabolic dysfunction or damage over disease may indicate still enough resilience to counteract dysfunction or damage from progressing to disease, a trait possibly related to participants’ young age. Consequently, there may be potential to enhance resilience through lifestyle changes or pharmacological treatments. If obesity is a model of accelerated aging, it could create opportunities for translational aging research and clinical trials focused on antiaging interventions.

### Strengths and Limitations

This study has strengths. To our knowledge, this MECC study is the first to explore how long-term obesity may be associated with early-onset aging in young adults. It examined a range of markers from cellular to systemic levels, considered significant developmental exposures, and integrated epidemiology, medicine, and geroscience.

However, some limitations should be considered when interpreting our results. First, observational cohorts inherently carry loss-to-follow-up bias. Yet, their main strength lies in the rich historical data, which was crucial for testing our hypothesis. Additionally, our research exhibited selection bias, as it was conducted on a nonrandom subset of the original cohort due to budget limitations. Recognizing the need for participant selection, we chose the design that best addresses bias and enhances data analysis efficiency.^32^ Second, a limitation of using BMI as the primary exposure is that it does not accurately represent body fat distribution or quantity.^59^ Yet, BMI outperformed other anthropometric and dual-energy x-ray absorptiometry–derived body composition markers in estimating participants’ epigenetic age (eTable 6 in Supplement 1); it also remains the most widely used obesity marker in large-scale settings. By using standardized BMI to track over-time trends, we also reduced inconsistencies in health risks associated with age, sex, and ethnicity. Further research should investigate how body composition influences obesity-induced accelerated aging. Third, although findings from Chilean young adults may not directly apply to other populations, they offer valuable insights into the association between aging and obesity in ethnically admixed, underserved communities exposed to obesogenic environments. As the cohort developed, Chile moved from low-income to high-income status, contributing to rising obesity.^60^ Studying a population affected by both epidemiological and economic transitions uniquely contributes to understanding how obesity may hasten the aging health consequences, a concern for rapidly developing low- and middle-income settings facing similar challenges. Fourth, we cannot definitively determine whether cardiometabolic disruption preceded the expression of aging markers, but our sample included 20 individuals with obesity without cardiometabolic comorbidities whose epigenetic age exceeded their chronological age (eTable 7 in Supplement 1). This supports the notion that obesity may trigger aging markers, disrupting homeostasis and leading to cardiometabolic dysfunction. Fifth, observational studies frequently indicate associations that should be validated through mechanistic studies or experiments. Nonetheless, insights about the effects of specific exposures often first arise from observations of affected individuals.^42^ Causality can be inferred from observational studies if certain criteria are met, like the Bradford Hill criteria; this framework remains the most referenced for causal inference in epidemiology and also when integrating data from molecular biology.^61^

## Conclusions

This MECC study, embedded in a prospective cohort, found that long-term obesity was associated with the emergence of molecular signals linked to primary, antagonistic, and integrative aging hallmarks in young adults. Obesity was associated with serious cardiometabolic abnormalities, potentially leading to early-onset cardiometabolic diseases. A research challenge is to determine how quickly cardiometabolic dysfunction progresses into disease in individuals with the obesity-induced accelerated aging phenotype, as such a diagnosis raises the multimorbidity risk. While this is established for middle-aged or older adults, how fast this occurs in relatively young adults needs further investigation. Since the early-aging phenotype was associated with the epigenome and participants were of reproductive age, future research should examine its potential inheritance.

## Notes

Supplement 1. **eFigure 1.** Flowchart of the Santiago Longitudinal Study, 1992-2024 **eTable 1.** Comparison of Included vs Excluded SLS Participants **eAppendix 1.** Expanding Upon the Differences Between Included vs Excluded Participants in the Study **eFigure 2.** BMI Trajectory From Birth to Adulthood in the Study Participants **eAppendix 2.** Methodological Note on BMI Assessment From Birth to Adulthood and Polynomial-Based Interpolation of BMI Trajectory **eTable 2.** Description of Clinical and Biochemical Procedures, Techniques, and References for Diagnosis of Cardiometabolic Risk **eTable 3.** Sample Description by Sex at 29-Year Assessment of Participants **eTable 4.** Prevalence of Cardiometabolic Comorbidities in the Sample by BMI Trajectory Across the Life Course **eFigure 3.** Changes in Raw BMI and Selected Cardiometabolic Markers From Adolescence to Adulthood by BMI Trajectory Across the Life Course **eAppendix 3.** Expanding Upon the Link Between Obesity and Aging-Related Epigenetic Changes **eTable 5.** Aging-Related Cytokines, Adipokines, Myokines, and Growth Factors in Participants by BMI Trajectory **eAppendix 4.** Expanding Upon Obesity-Related Cytokines, Adipokines, Myokines, and Growth Factors Profile as Molecular Aging Signatures **eAppendix 5.** Expanding Upon the Clinical Implications of the Findings **eTable 6.** Epigenetic Aging-Related Profile in Participants With Long-Term Obesity and Free of Cardiometabolic Comorbidities **eTable 7.** Correlation Between Sex-Adjusted Anthropometric and DXA-Derived Body Composition Markers With Epigenetic Age in Participants
eReferences

Supplement 2.
Data Sharing Statement

## References

[1] Khan SS, Ning H, Wilkins JT, . Association of body mass index with lifetime risk of cardiovascular disease and compression of morbidity. JAMA Cardiol. 2018;3(4):280-287. doi:10.1001/jamacardio.2018.0022 29490333 PMC5875319

[2] Iyen B, Weng S, Vinogradova Y, Akyea RK, Qureshi N, Kai J. Long-term body mass index changes in overweight and obese adults and the risk of heart failure, cardiovascular disease and mortality: a cohort study of over 260,000 adults in the UK. BMC Public Health. 2021;21(1):576. doi:10.1186/s12889-021-10606-1 33853578 PMC8048253

[3] Friedenreich CM, Ryder-Burbidge C, McNeil J. Physical activity, obesity and sedentary behavior in cancer etiology: epidemiologic evidence and biologic mechanisms. Mol Oncol. 2021;15(3):790-800. doi:10.1002/1878-0261.12772 32741068 PMC7931121

[4] Cotangco KR, Liao CI, Eakin CM, . Trends in incidence of cancers associated with obesity and other modifiable risk factors among women, 2001-2018. Prev Chronic Dis. 2023;20:E21. doi:10.5888/pcd20.220211 36996404 PMC10109476

[5] Chen N, Fong DYT, Wong JYH. Health and economic outcomes associated with musculoskeletal disorders attributable to high body mass index in 192 countries and territories in 2019. JAMA Netw Open. 2023;6(1):e2250674. doi:10.1001/jamanetworkopen.2022.50674 36662529 PMC9860530

[6] Cizza G, Brown RJ, Rother KI. Rising incidence and challenges of childhood diabetes: a mini review. J Endocrinol Invest. 2012;35(5):541-546. 22572768 10.3275/8411 PMC3485685

[7] Nadeau KJ, Anderson BJ, Berg EG, . Youth-onset type 2 diabetes consensus report: current status, challenges, and priorities. Diabetes Care. 2016;39(9):1635-1642. doi:10.2337/dc16-1066 27486237 PMC5314694

[8] Kim BC, Kim MK, Han K, . Low muscle mass is associated with metabolic syndrome only in nonobese young adults: the Korea National Health and Nutrition Examination Survey 2008-2010. Nutr Res. 2015;35(12):1070-1078. doi:10.1016/j.nutres.2015.09.020 26602833

[9] Burrows R, Correa-Burrows P, Reyes M, Blanco E, Albala C, Gahagan S. Low muscle mass is associated with cardiometabolic risk regardless of nutritional status in adolescents: a cross-sectional study in a Chilean birth cohort. Pediatr Diabetes. 2017;18(8):895-902. doi:10.1111/pedi.12505 28145023 PMC5538898

[10] Marinac CR, Birmann BM. Rising cancer incidence in younger adults: is obesity to blame? Lancet Public Health. 2019;4(3):e119-e120. doi:10.1016/S2468-2667(19)30017-9 30733055

[11] Salvestrini V, Sell C, Lorenzini A. Obesity may accelerate the aging process. Front Endocrinol (Lausanne). 2019;10:266. doi:10.3389/fendo.2019.00266 31130916 PMC6509231

[12] Tam BT, Morais JA, Santosa S. Obesity and ageing: two sides of the same coin. Obes Rev. 2020;21(4):e12991. doi:10.1111/obr.12991 32020741

[13] López-Otín C, Blasco MA, Partridge L, Serrano M, Kroemer G. Hallmarks of aging: an expanding universe. Cell. 2023;186(2):243-278. doi:10.1016/j.cell.2022.11.001 36599349

[14] Di Angelantonio E, Bhupathiraju ShN, Wormser D, ; Global BMI Mortality Collaboration. Body-mass index and all-cause mortality: individual-participant-data meta-analysis of 239 prospective studies in four continents. Lancet. 2016;388(10046):776-786. doi:10.1016/S0140-6736(16)30175-1 27423262 PMC4995441

[15] Flegal KM, Kit BK, Orpana H, Graubard BI. Association of all-cause mortality with overweight and obesity using standard body mass index categories: a systematic review and meta-analysis. JAMA. 2013;309(1):71-82. doi:10.1001/jama.2012.113905 23280227 PMC4855514

[16] Berrington de Gonzalez A, Hartge P, Cerhan JR, . Body-mass index and mortality among 1.46 million White adults. N Engl J Med. 2010;363(23):2211-2219. doi:10.1056/NEJMoa1000367 21121834 PMC3066051

[17] Reilly JJ, Kelly J. Long-term impact of overweight and obesity in childhood and adolescence on morbidity and premature mortality in adulthood: systematic review. Int J Obes (Lond). 2011;35(7):891-898. doi:10.1038/ijo.2010.222 20975725

[18] Fülöp T, Larbi A, Witkowski JM. Human inflammaging. Gerontology. 2019;65(5):495-504. doi:10.1159/000497375 31055573

[19] Ley RE, Bäckhed F, Turnbaugh P, Lozupone CA, Knight RD, Gordon JI. Obesity alters gut microbial ecology. Proc Natl Acad Sci U S A. 2005;102(31):11070-11075. doi:10.1073/pnas.0504978102 16033867 PMC1176910

[20] Zhou Y, Hambly BD, McLachlan CS. FTO associations with obesity and telomere length. J Biomed Sci. 2017;24(1):65. doi:10.1186/s12929-017-0372-6 28859657 PMC5580219

[21] de Mello AH, Costa AB, Engel JDG, Rezin GT. Mitochondrial dysfunction in obesity. Life Sci. 2018;192:26-32. doi:10.1016/j.lfs.2017.11.019 29155300

[22] Wen X, Zhang B, Wu B, . Signaling pathways in obesity: mechanisms and therapeutic interventions. Signal Transduct Target Ther. 2022;7(1):298. doi:10.1038/s41392-022-01149-x 36031641 PMC9420733

[23] Bartelt A, Widenmaier SB. Proteostasis in thermogenesis and obesity. Biol Chem. 2020;401(9):1019-1030. doi:10.1515/hsz-2019-0427 32061163

[24] Newsholme P, de Bittencourt PI Jr. The fat cell senescence hypothesis: a mechanism responsible for abrogating the resolution of inflammation in chronic disease. Curr Opin Clin Nutr Metab Care. 2014;17(4):295-305. doi:10.1097/MCO.0000000000000077 24878874

[25] Franceschi C. Healthy ageing in 2016: obesity in geroscience—is cellular senescence the culprit? Nat Rev Endocrinol. 2017;13(2):76-78. doi:10.1038/nrendo.2016.213 28059157

[26] Horvath S, Erhart W, Brosch M, . Obesity accelerates epigenetic aging of human liver. Proc Natl Acad Sci U S A. 2014;111(43):15538-15543. doi:10.1073/pnas.1412759111 25313081 PMC4217403

[27] de Toro-Martín J, Guénard F, Tchernof A, . Body mass index is associated with epigenetic age acceleration in the visceral adipose tissue of subjects with severe obesity. Clin Epigenetics. 2019;11(1):172. doi:10.1186/s13148-019-0754-6 31791395 PMC6888904

[28] Lobstein T, Brindsen H. Obesity: Missing the 2025 Global Targets—Trends, Costs and Country Reports. World Obesity Federation; 2020. Accessed June 6, 2024. https://s3-eu-west-1.amazonaws.com/wof-files/970_-_WOF_Missing_the_2025_Global_Targets_Report_ART.pdf

[29] Lozoff B, De Andraca I, Castillo M, Smith JB, Walter T, Pino P. Behavioral and developmental effects of preventing iron-deficiency anemia in healthy full-term infants. Pediatrics. 2003;112(4):846-854. doi:10.1542/peds.112.4.846 14523176

[30] Correa-Burrows P, Rogan J, Blanco E, . Resolving early obesity leads to a cardiometabolic profile within normal ranges at 23 years old in a two-decade prospective follow-up study. Sci Rep. 2021;11(1):18927. doi:10.1038/s41598-021-97683-9 34556688 PMC8460734

[31] Correa-Burrows P, Burrows R, Albala C, . Multiple events case-control study in a prospective cohort to identify systemic, cellular, and molecular biomarkers of obesity-induced accelerated aging in 30-years-olds: the ObAGE study protocol. BMC Geriatr. 2022;22(1):387. doi:10.1186/s12877-022-03032-4 35501766 PMC9063300

[32] Sun W, Joffe MM, Chen J, Brunelli SM. Design and analysis of multiple events case-control studies. Biometrics. 2010;66(4):1220-1229. doi:10.1111/j.1541-0420.2009.01369.x 20002403 PMC2980800

[33] de Onis M, Onyango AW, Borghi E, Siyam A, Nishida C, Siekmann J. Development of a WHO growth reference for school-aged children and adolescents. Bull World Health Organ. 2007;85(9):660-667. doi:10.2471/BLT.07.043497 18026621 PMC2636412

[34] Emery W, Thompson R. Statistical methods and error handling. In: Data Analysis Methods in Physical Oceanography. Elsevier; 2001:193-304. doi:10.1016/B978-044450756-3/50004-6

[35] Pollock D. Smoothing with cubic splines. In: Handbook of Time Series Analysis, Signal Processing, and Dynamics. Academic Press; 1999:293-322. doi:10.1016/B978-012560990-6/50013-0

[36] Horvath S. DNA methylation age of human tissues and cell types. Genome Biol. 2013;14(10):R115. doi:10.1186/gb-2013-14-10-r115 24138928 PMC4015143

[37] Lu AT, Quach A, Wilson JG, . DNA methylation GrimAge strongly predicts lifespan and healthspan. Aging (Albany NY). 2019;11(2):303-327. doi:10.18632/aging.101684 30669119 PMC6366976

[38] Alberti KG, Eckel RH, Grundy SM, ; International Diabetes Federation Task Force on Epidemiology and Prevention; National Heart, Lung, and Blood Institute; American Heart Association; World Heart Federation; International Atherosclerosis Society; International Association for the Study of Obesity. Harmonizing the metabolic syndrome: a joint interim statement of the International Diabetes Federation Task Force on Epidemiology and Prevention; National Heart, Lung, and Blood Institute; American Heart Association; World Heart Federation; International Atherosclerosis Society; and International Association for the Study of Obesity. Circulation. 2009;120(16):1640-1645. doi:10.1161/CIRCULATIONAHA.109.192644 19805654

[39] DeBoer MD, Gurka MJ. Clinical utility of metabolic syndrome severity scores: considerations for practitioners. Diabetes Metab Syndr Obes. 2017;10:65-72. doi:10.2147/DMSO.S101624 28255250 PMC5325095

[40] Hamaguchi M, Kojima T, Itoh Y, . The severity of ultrasonographic findings in nonalcoholic fatty liver disease reflects the metabolic syndrome and visceral fat accumulation. Am J Gastroenterol. 2007;102(12):2708-2715. doi:10.1111/j.1572-0241.2007.01526.x 17894848

[41] Gibbons R, Hedeker D, Davis J. Estimation of effect size from a series of experiments involving paired comparisons. J Educ Stat. 1993;18(3):271-279. doi:10.3102/10769986018003271

[42] Gianicolo EAL, Eichler M, Muensterer O, Strauch K, Blettner M. Methods for evaluating causality in observational studies. Dtsch Arztebl Int. 2020;116(7):101-107. doi:10.3238/arztebl.2020.0101 32164822 PMC7081045

[43] Oblak L, van der Zaag J, Higgins-Chen AT, Levine ME, Boks MP. A systematic review of biological, social and environmental factors associated with epigenetic clock acceleration. Ageing Res Rev. 2021;69:101348. doi:10.1016/j.arr.2021.101348 33930583

[44] López-Otín C, Blasco MA, Partridge L, Serrano M, Kroemer G. The hallmarks of aging. Cell. 2013;153(6):1194-1217. doi:10.1016/j.cell.2013.05.039 23746838 PMC3836174

[45] Pedersen L, Hojman P. Muscle-to-organ cross talk mediated by myokines. Adipocyte. 2012;1(3):164-167. doi:10.4161/adip.20344 23700527 PMC3609091

[46] Severinsen MCK, Pedersen BK. Muscle-organ crosstalk: the emerging roles of myokines. Endocr Rev. 2020;41(4):594-609. doi:10.1210/endrev/bnaa016 32393961 PMC7288608

[47] Chen ZT, Weng ZX, Lin JD, Meng ZX. Myokines: metabolic regulation in obesity and type 2 diabetes. Life Metab. 2024;3(3):loae006. doi:10.1093/lifemeta/loae006 39872377 PMC11749576

[48] Fang P, She Y, Yu M, Min W, Shang W, Zhang Z. Adipose-Muscle crosstalk in age-related metabolic disorders: The emerging roles of adipo-myokines. Ageing Res Rev. 2023;84:101829. doi:10.1016/j.arr.2022.101829 36563906

[49] Jia J, Yu F, Wei WP, . Relationship between circulating irisin levels and overweight/obesity: a meta-analysis. World J Clin Cases. 2019;7(12):1444-1455. doi:10.12998/wjcc.v7.i12.1444 31363472 PMC6656672

[50] Li C, Cheng H, Adhikari BK, . The role of apelin-APJ system in diabetes and obesity. Front Endocrinol (Lausanne). 2022;13:820002. doi:10.3389/fendo.2022.820002 35355561 PMC8959308

[51] González-Gualda E, Baker AG, Fruk L, Muñoz-Espín D. A guide to assessing cellular senescence in vitro and in vivo. FEBS J. 2021;288(1):56-80. doi:10.1111/febs.15570 32961620

[52] Zhang WB, Ye K, Barzilai N, Milman S. The antagonistic pleiotropy of insulin-like growth factor 1. Aging Cell. 2021;20(9):e13443. doi:10.1111/acel.13443 34363732 PMC8441393

[53] Gubbi S, Quipildor GF, Barzilai N, Huffman DM, Milman S. 40 years of IGF1: IGF1: the Jekyll and Hyde of the aging brain. J Mol Endocrinol. 2018;61(1):T171-T185. doi:10.1530/JME-18-0093 29739805 PMC5988994

[54] Muhammad T, Wan Y, Sha Q, . IGF2 improves the developmental competency and meiotic structure of oocytes from aged mice. Aging (Albany NY). 2020;13(2):2118-2134. doi:10.18632/aging.202214 33318299 PMC7880328

[55] Zhou X, Tan B, Gui W, . IGF2 deficiency promotes liver aging through mitochondrial dysfunction and upregulated CEBPB signaling in D-galactose-induced aging mice. Mol Med. 2023;29(1):161. doi:10.1186/s10020-023-00752-0 38017373 PMC10685569

[56] Burtscher J, Soltany A, Visavadiya NP, . Mitochondrial stress and mitokines in aging. Aging Cell. 2023;22(2):e13770. doi:10.1111/acel.13770 36642986 PMC9924952

[57] Burrows R, Correa-Burrows P, Rogan J, Cheng E, Blanco E, Gahagan S. Long-term vs recent-onset obesity: their contribution to cardiometabolic risk in adolescence. Pediatr Res. 2019;86(6):776-782. doi:10.1038/s41390-019-0543-0 31426054 PMC6891158

[58] Mattsson M, Maher GM, Boland F, Fitzgerald AP, Murray DM, Biesma R. Group-based trajectory modelling for BMI trajectories in childhood: a systematic review. Obes Rev. 2019;20(7):998-1015. doi:10.1111/obr.12842 30942535

[59] The science, strengths, and limitations of body mass index. In: Callahan EA, ed. Translating Knowledge of Foundational Drivers of Obesity Into Practice: Proceedings of a Workshop Series. National Academies Press; 2023:chap 10. Accessed June 28, 2024. https://www.ncbi.nlm.nih.gov/books/NBK594362/37639520

[60] Albala C, Vio F, Kain J, Uauy R. Nutrition transition in Chile: determinants and consequences. Public Health Nutr. 2002;5(1A):123-128. doi:10.1079/PHN2001283 12027274

[61] Fedak KM, Bernal A, Capshaw ZA, Gross S. Applying the Bradford Hill criteria in the 21st century: how data integration has changed causal inference in molecular epidemiology. Emerg Themes Epidemiol. 2015;12:14. doi:10.1186/s12982-015-0037-4 26425136 PMC4589117

