# Supplementary material for: Long-Term Obesity and Biological Aging in Young Adults
Source: JAMA Netw Open. 2025 Jul 11;8(7):e2520011. doi: 10.1001/jamanetworkopen.2025.20011 (PMC12254895; doi:10.1001/jamanetworkopen.2025.20011)
Supplement: Supplement 1. — eFigure 1. Flowchart of the Santiago Longitudinal Study, 1992-2024 eTable 1. Comparison of Included vs Excluded SLS Participants eAppendix 1. Expanding Upon the Differences Between Included vs Excluded Participants in the Study eFigure 2. BMI Trajectory From Birth to Adulthood in the Study Participants eAppendix 2. Methodological Note on BMI Assessment From Birth to Adulthood and Polynomial-Based Interpolation of BMI Trajectory eTable 2. Description of Clinical and Biochemical Procedures, Techniques, and References for Diagnosis of Cardiometabolic Risk eTable 3. Sample Description by Sex at 29-Year Assessment of Participants eTable 4. Prevalence of Cardiometabolic Comorbidities in the Sample by BMI Trajectory Across the Life Course eFigure 3. Changes in Raw BMI and Selected Cardiometabolic Markers From Adolescence to Adulthood by BMI Trajectory Across the Life Course eAppendix 3. Expanding Upon the Link Between Obesity and Aging-Related Epigenetic Changes eTable 5. Aging-Related Cytokines, Adipokines, Myokines, and Growth Factors in Participants by BMI Trajectory eAppendix 4. Expanding Upon Obesity-Related Cytokines, Adipokines, Myokines, and Growth Factors Profile as Molecular Aging Signatures eAppendix 5. Expanding Upon the Clinical Implications of the Findings eTable 6. Epigenetic Aging-Related Profile in Participants With Long-Term Obesity and Free of Cardiometabolic Comorbidities eTable 7. Correlation Between Sex-Adjusted Anthropometric and DXA-Derived Body Composition Markers With Epigenetic Age in Participants eReferences [file jamanetwopen-e2520011-s001.pdf]

Supplementary Online Content

Correa-Burrows P, Burrows R, Albala C, et al. Long-term obesity and biological aging in young adults. *JAMA Netw Open*. 2025;8(7):e2520011. doi:10.1001/jamanetworkopen.2025.20011

eFigure 1. Flowchart of the Santiago Longitudinal Study, 1992-2024

eTable 1. Comparison of Included vs Excluded SLS Participants

eAppendix 1. Expanding Upon the Differences Between Included vs Excluded Participants in the Study

eFigure 2. BMI Trajectory From Birth to Adulthood in the Study Participants

eAppendix 2. Methodological Note on BMI Assessment From Birth to Adulthood and Polynomial-Based Interpolation of BMI Trajectory

eTable 2. Description of Clinical and Biochemical Procedures, Techniques, and References for Diagnosis of Cardiometabolic Risk

eTable 3. Sample Description by Sex at 29-Year Assessment of Participants

eTable 4. Prevalence of Cardiometabolic Comorbidities in the Sample by BMI Trajectory Across the Life Course

eFigure 3. Changes in Raw BMI and Selected Cardiometabolic Markers From Adolescence to Adulthood by BMI Trajectory Across the Life Course

eAppendix 3. Expanding Upon the Link Between Obesity and Aging-Related Epigenetic Changes

eTable 5. Aging-Related Cytokines, Adipokines, Myokines, and Growth Factors in Participants by BMI Trajectory

eAppendix 4. Expanding Upon Obesity-Related Cytokines, Adipokines, Myokines, and Growth Factors Profile as Molecular Aging Signatures

eAppendix 5. Expanding Upon the Clinical Implications of the Findings

eTable 6. Epigenetic Aging-Related Profile in Participants With Long-Term Obesity and Free of Cardiometabolic Comorbidities

eTable 7. Correlation Between Sex-Adjusted Anthropometric and DXA-Derived Body Composition Markers With Epigenetic Age in Participants

eReferences

This supplementary material has been provided by the authors to give readers additional information about their work.

© 2025 Correa-Burrows P et al. *JAMA Network Open*.

eFigure 1 Flowchart of the Santiago Longitudinal Study, 1992-2024

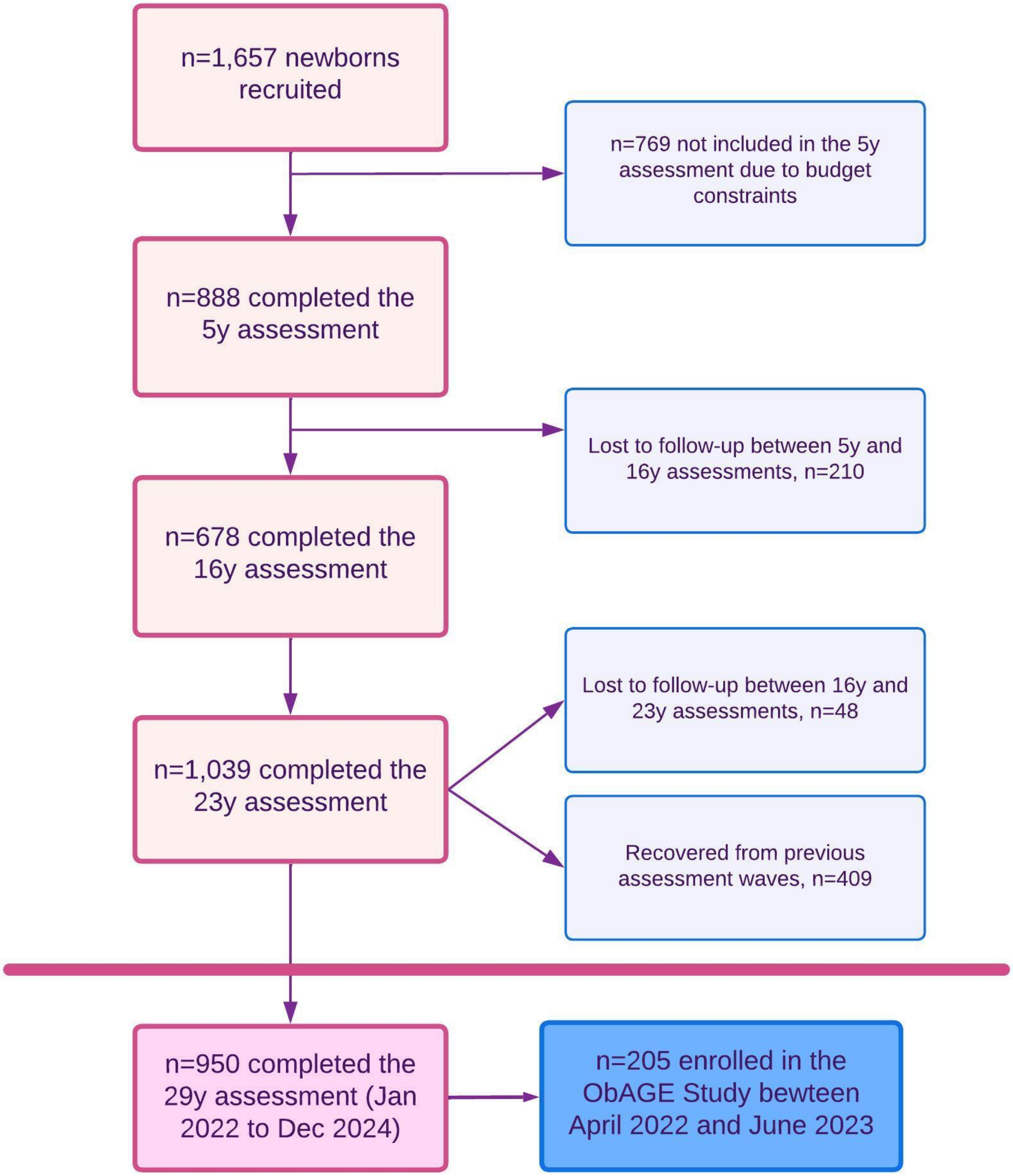

The Santiago Longitudinal Study (SLS), Chile's oldest epidemiological birth cohort, consists of females and males of low- to middle socioeconomic status, from the Southeast neighborhoods of Santiago. Participants joined the cohort as newborns to participate in research related to nutrition and development as infants with follow-up at 1y, 5y, 10y, 12y, 14y, 16y, and 23y. They were born in 1992-1996, at term, of uncomplicated vaginal births, weighed >3.0 kg, and were free of acute or chronic health problems [1]. Enrollment occurred just as the country entered a period of rapid socioeconomic changes associated with decreasing undernutrition and infectious disease and increasing NCD [2,3]. In 2009, when the participants were 16-17y, they were invited to get involved in an NIH-funded study of 'Biopsychosocial Determinants of Adolescent Obesity and Cardiovascular Risk.' Assessment of anthropometric and cardiometabolic markers was repeated at 23y, and again at 29y. In the 29-year assessment phase, the Santiago Longitudinal Study (SLS) enrolled 950

© 2025 Correa-Burrows P et al. *JAMA Network Open*.

participants, with a selected group taking part in the 'Obesity-induced Accelerated Aging Study (ObAGE)' due to financial constraints associated with the expensive molecular profiling central to ObAGE. Hence, ObAGE was conceived as a multiple-event case-control (MECC) study embedded in the prospective SLS. In the MECC design, there is a known and enumerated cohort, from which subjects are selected for additional measurements. Sun et al. demonstrated that MECC is superior to case-cohort or nested case-control studies as the former eliminates bias and improves the efficiency of the same data analysis [4]. The ObAGE subset was randomly selected among participants with: 1) complete data in all assessment waves (birth, 1, 5, 10, 12, 14, 16, 23, 29y) to maximize the accuracy of the spline algorithm estimating the BMI trajectories, the main exposure in the proposed study; and 2) falling into one of the following BMI trajectory group: Group 1: participants always having a BMI in the healthy range; Group 2: participants with obesity starting in adolescence and remaining obese into adulthood; Group 3: participants who were obese in early childhood and remained obese into adulthood.

© 2025 Correa-Burrows P et al. *JAMA Network Open*.

eTable 1 Comparison of included vs. excluded SLS participants

	ObAGE Participants (n=205)		Non-ObAGE Participants (n=526)		P value[a]
	Mean | Median	SD | IQR	Mean | Median	SD | IQR	
Chronological age (y)	28.9	0.7	28.9	0.8	.09
Sex (male | n and %)	105	51.2%	164	50.6%	.08[b]
Body-mass Index	31.5	7.42	29.2	6.33	<.001
Waist circumference (cm) - males	96.7	15.1	92.5	11.1	<.05
Waist circumference (cm) - females	90.1	15.9	87.6	12.9	.07
Waist-to-height ratio - males	0.56	.08	0.53	.06	<.01
Waist-to-height ratio - females	0.56	.09	0.55	.07	.07
Systolic BP (mm Hg)	120	(111-128)	118	(110-125)	<.05[c]
Diastolic BP (mm Hg)	76	(69-82)	75	(69-81)	.49[c]
Fasting glycemia (mg/dL)	93.9	21.2	92.7	16.9	.25
Fasting insulin (uUI/L)	12.4	(8.5-17.1)	11.4	(8.1-14.9)	.14[c]
HOMA-IR	2.9	(1.8-4.0)	2.6	(1.7-3.5)	.17[c]
Fasting triglycerides (mg/dL)	95.8	(62.5-149.8)	88.9	(63.9-135)	.22[c]
Total cholesterol (mg/dL)	176.8	(151.4-201)	175.5	(153.1-199)	.69[c]
HDL cholesterol (mg/dL) - males	20.3	(16.7-26.2)	23.5	(18.6-29.7)	<.01[c]
HDL cholesterol (mg/dL) - females	24.6	(19.0-30.0)	26.1	(20.9-34.7)	<.05
hs CRP (mg/L)	2.4	(0-8-4.6)	1.7	(0-7-3.6)	<.01[c]
**Weight status**					
Obesity	116	56.5%	170	32.3%	<.001[b]
**Cardiometabolic-related alterations**					
Insulin resistance	118	54.6%	270	51.3%	.39[b]
Metabolic syndrome	114	36.8%	167	31.8%	.13[b]
LGS inflammation	86	42.2%	166	31.6%	<.01[b]
Type-2 diabetes mellitus	1	0.48%	4	0.76%	1.0[d]
Metformin (currently taking)	4	1.95%	19	3.61%	.42[d]

Values are expressed as mean and SD, median and (IQR) or n and percentage. SLS participants not taking part in the ObAGE study also include males and females with overweight. [a] Two-tailed Student's t test unless otherwise indicated. [b]Pearson's Chi2 test for independence. [c]Wilcoxson-Mann-Whitney test. [d]Two-tailed Fisher's exact test. Insulin Resistance: HOMA-IR ≥2.6; Metabolic Syndrome: AHA/IDF/NBHLI Joint definition; LGS inflammation: Low-grade systemic inflammation: hs-CRP >3 mg/L.

© 2025 Correa-Burrows P et al. *JAMA Network Open*.

eAppendix 1 Expanding upon the differences between included vs excluded participants in the study

The Obesity-induced accelerated aging (ObAGE) Study was conceived as a multiple events case-control (MECC) study embedded in a prospective cohort. In the MECC design, there is a known and enumerated cohort, from which subjects are selected for additional measurements. Sun et al. demonstrated that MECC is superior to case-cohort or nested case-control studies as the former eliminates bias and improves the efficiency of the same data analysis [4]. We included participants with complete data in all assessment waves who fell into one of the following categories of lifetime BMI trajectory: (1) participants always having a BMI in the healthy range (TG1); (2) participants with obesity starting in adolescence and remained obese into adulthood (TG2); (3) participants who were obese in early childhood and remained obese into adulthood (TG3). Between April 2022 and June 2023, we enrolled n=205 participants. This sample size allows testing the study hypotheses and detecting moderate to small differences (f=0.15) at α=0.05 and 1-β=0.8. The subset included in this study comprised 52% of subjects with obesity compared to 32% of participants with obesity in participants not meeting the criteria to enter the ObAGE study. In the ObAGE group, the mean BMI exceeded that of the non-ObAGE group, yet both groups had BMIs well above the healthy threshold. Similarly, among ObAGE men, waist circumference (WC) and waist-to-height ratio (WtHT) surpassed those of non-ObAGE men; yet, again both groups exhibited mean values above the healthy standards. No differences were noted in WC and WtHT among women. The median systolic blood pressure in the ObAGE group was higher than in the non-ObAGE group, just at the elevated blood pressure cutoff. In both sexes, median HDL levels were reduced in ObAGE individuals vs non-ObAGE individuals, yet the latter also recorded values far below the recommended thresholds for maintaining cardiometabolic health. Also, hsCRP levels were higher in the ObAGE group than in the non-ObAGE group. No differences were found between ObAGE and non-ObAGE subjects for other cardiometabolic variables. Thus, while ObAGE comprises a subset of the SLS with a higher number of obese individuals owing to the study's design, the differences in cardiometabolic profiles between the two groups are limited. Where differences do occur, they were of small magnitude.

© 2025 Correa-Burrows P et al. *JAMA Network Open*.

eFigure 2 BMI trajectory from birth to adulthood in the study participants

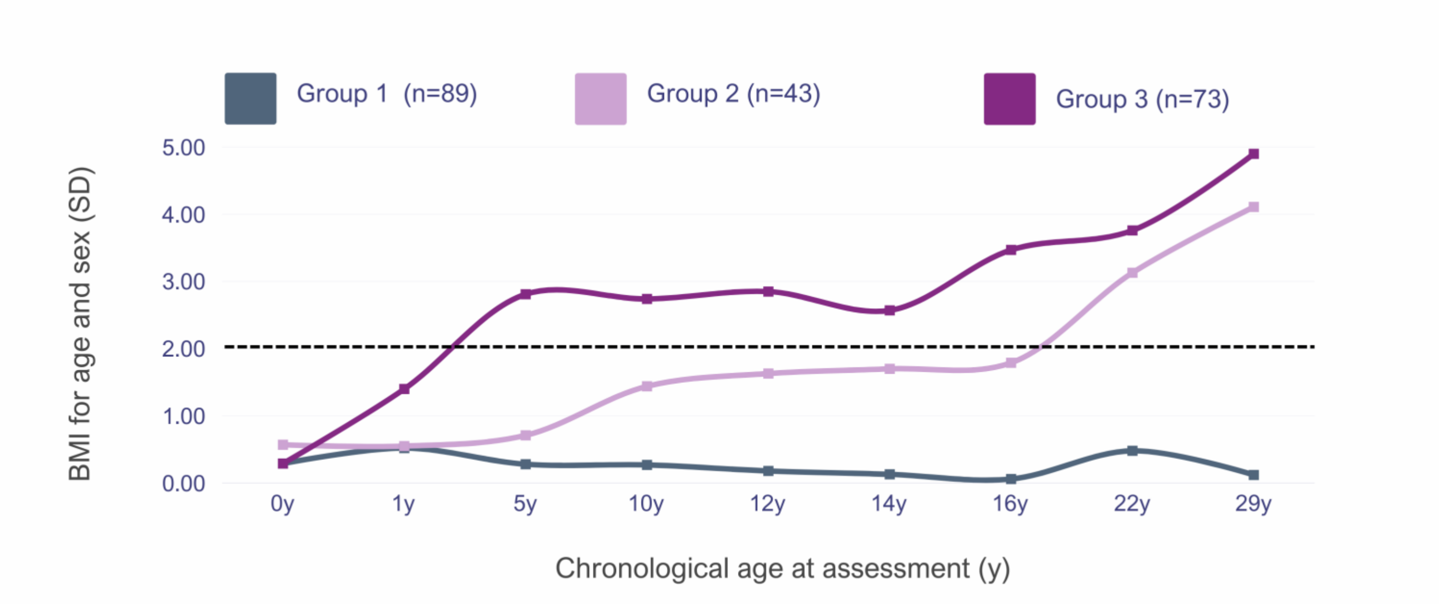

Polynomial-based interpolation of BMI trajectory from birth to adulthood. BMI estimated from weight (kg) and height (cm) measured at several time points was standardized (z score or BMI for age and sex) according to WHO references. Reference values for males and females 19 years and older were used to standardize BMI assessed in adulthood. Group 1: participants always having a BMI in the healthy range; Group 2: participants with obesity starting in adolescence and remaining obese into adulthood; Group 3: participants who were obese in early childhood and remained obese into adulthood. Obesity was diagnosed with a BMI z score ≥ 2.0 SD (dotted black line in the diagram).

© 2025 Correa-Burrows P et al. *JAMA Network Open*.

eAppendix 2 Methodological note on BMI assessment (birth to adulthood) and polynomial-based interpolation of BMI trajectory

BMI estimated from weight (kg) and height (cm) measured at several time points from birth to adulthood was standardized (z score) according to the 2006 and 2007 WHO growth references [5,6]. Reference values for males and females 19 years and older were used to standardize BMI assessed in adulthood. We used a cubic polynomial spline to interpolate each participant's BMI trajectory from birth to early adulthood. This method takes the data points from original measurements, and the splines smooth the transition between data points. Spline interpolation is often favored over other polynomial interpolation methods (such as Lagrange and Newton polynomials) because it can be used for both segments and entire data series. It also allows for small interpolation errors even when using low-degree polynomials for the spline [8]. The second advantage of this method is that it allows the construction of a smooth and visually pleasing parametric curve when dealing with sparse data [7], particularly if the spline departs from the original data points [8], as was the case here (we had data for birthweight in all participants). Thus, spline interpolation is more consistent with how BMI changes over time than linear approaches, in which two data points are connected through a straight line. Linear interpolation uses a linear function for each interval, and although quick and easy, it is not very precise. On the other hand, Spline interpolation uses low-degree polynomials in each interval and chooses the polynomial pieces to fit smoothly together [7,8]. Following the same method described in Correa et al. [9], we fitted models with data measured at birth, 1 year, 5 years, 10 years, 12 years, 14 years, 16 years, 21 years, 23 years, and 28-29 years and obtained individual BMI trajectories from birth to adulthood. Using the full BMI trajectories available, we estimated the timing of obesity onset and duration in those participants who ever had obesity, with a precision of weeks. Python 3.0 was used for data interpolation and BMI trajectory modeling.

© 2025 Correa-Burrows P et al. *JAMA Network Open*.

eTable 2 Description of clinical and biochemical procedures, techniques, and references for diagnosis of cardiometabolic risk.

Measurement	Method	Description
Blood pressure	Oscillometric technique	Three blood pressure (BP) measurements were made, with the participant at rest seated for at least 15 minutes. An individual was defined as being normotensive when presenting with systolic blood pressure (SBP) < 120 mmHg and diastolic blood pressure (DBP) < 85 mmHg. In all cases, BP was measured at the upper arm using an OMRON 705IT oscillometric monitor following international and national guidelines [10].
Pulse-wave velocity	Oscillometric technique	Aortic pulse-wave (PWV)(m/s^2) velocity, one of the most important clinical parameters for evaluating cardiovascular risk and vascular adaptation, was measured with an oscillometric technique using the Arteriograph system (TecnoMed, Madrid, Spain). This device measures the pulse wave at a single location using a brachial cuff inflated to 35 mmHg over the SBP. The time between the first systolic wave and the second reflected wave is measured as transit time. To calculate pulse wave velocity, the device uses the ratio of the traveled distance between the jugulum and symphysis to the transit time. Reference values of PWV were those estimated for Hispanic populations [11]. Values >75th percentile were considered arterial stiffness.
Fasting glucose	Colorimetric analysis	Glucose (mg/dl) was measured with an enzymatic colorimetric test (QCA SA, Amposta, Spain). Values >100 mg/dl were considered fasting hyperglycemia.
Fasting insulin	RIA	Radioimmunoassay (Diagnostic Products Corporation, Los Angeles, CA) was used to determine insulin (uUI/l). Values >12 uUI/L were considered fasting hyperinsulinemia.
Lipid panel	Dry analytical method	Dry analytical methodology (Vitros; Ortho Clinical Diagnostics Inc, Raritan, NJ) was used to determine total cholesterol (mg/dl), triglycerides (mg/dl), and HDL cholesterol (mg/dl). Values >200 md/dl and >150 mg/dl were considered high total cholesterol and hypertriglyceridemia, respectively. HDL-chol <40 md/dl in males and <50 md/dl in females were considered below clinical ranges.
hs C-reactive protein	Sensitive latex-based immunoassay	Serum hs-CRP (mg/l) was measured with a sensitive latex-based immunoassay, and values of >1.0 mg/l were considered low-grade systemic inflammation, according to the AHA/CDC Joint Statement on Markers of Inflammation and Cardiovascular Disease [12]. To avoid abnormally high levels of hs-CRP (denoting acute inflammation), participants being ill (e.g., cold, viral infections, diarrhea, etc.) at least ten days before the evaluation had their appointment rescheduled.
Homeostatic Model Assessment	N/A	The homeostatic model assessment (HOMA-IR) quantified insulin sensitivity, with values ≥2.6 denoting insulin resistance (IR) [13]. We also estimated HOMA-β to approach the functioning of the pancreatic β-cell.
Metabolic Syndrome	N/A	Metabolic Syndrome was diagnosed according to the 2009 AHA/NHLBI/IDF Joint Interim Statement [14].
Metabolic Syndrome severity score	N/A	According to Gurka et al., a continuous Metabolic Syndrome severity risk score was computed, with values ≥ 1 SD considered high risk [15].
Assessment of the liver	Ultrasound	An abdominal ultrasound was performed on SLS participants using a General Electric LogiQ ultra-sonographer with a 4C RS convex multifrequency probe (2–5.5 MHz) (GE Healthcare Systems,

© 2025 Correa-Burrows P et al. *JAMA Network Open*.

		Wauwatosa, WI). All examinations were done by the same operator, who obtained and stored the images to be analyzed by two independent observers. The operator had training to obtain standardized images in which the liver and the right kidney were seen simultaneously. Images were obtained with the participant rolled onto its left side in a decubitus position, with the right arm stretched above the head after taking a deep breath. Observers were gastroenterologists with training in abdominal ultrasound interpretation and scored liver brightness (0-3), diaphragm attenuation (0-2), and vessel blurring (0-1), according to Hamaguchi et al. [16]. The maximum score possible is six. According to the validity assessment of this semi-quantitative method to determine liver fat infiltration, conducted in the SLS with Magnetic Resonance Spectroscopy (MRS) as the standard, values ≥4 have good sensitivity (82%) and specificity (84%) for non-alcoholic fatty liver disease diagnosis [17].
Carotid intima-media thickness	Ultrasound	During the examination, each patient was lying down with their head tilted to the side (recumbent) and the neck fully exposed. A General Electric LogiQ ultra-sonographer with a 4C RS convex multifrequency probe (2–5.5 MHz) (GE Healthcare Systems, Wauwatosa, WI) was used to scan the neck. The probe was placed on the neck and moved upwards along the anterior/posterior edge of the sternocleidomastoid muscle. Two-dimensional images of the transverse and longitudinal axes were selected to observe the external and internal carotid arteries. The scan covered the transverse and longitudinal axes of the extracranial segment from the internal carotid artery to the common carotid artery. The examination determined the presence of plaque in the carotid artery tube, the location and size of the plaque, blood vessel stenosis, and blood flow. The technique and classification of CIMT follow the standards outlined in the consensus statement from the American Society of Echocardiography (ASE) Carotid Intima-Media Thickness Task Force [18]. Age, sex, and ethnicity were considered while determining the 75th percentile of risk, consistent with the ASE CIMT Task Force. If the CIMT >75th percentile or if a carotid plaque is present, patients are at high risk of developing a cardiovascular event.
Body composition	DXA	A body composition analysis was conducted using a dual-energy X-ray absorptiometry (DXA) machine (Lunar Prodigy Corp., Madison, General Electric, WI), along with Lunar iDXA ENCORE 2011 software (Version 13.60.033 Copyright © 1998-2010). The scan was performed in the fasting state to determine the amount and percentage of body fat and lean mass in various body parts such as arms, legs, trunk, and the total body.

© 2025 Correa-Burrows P et al. *JAMA Network Open*.

eTable2 Description of statistical techniques used in this study

Overall description	Statistical analyses were conducted using Stata for Windows v.16.0 and XLSTAT-R, an interface between XLSTAT and the open-source R software. Data were expressed as mean (SD) or median (25th p–75th p) for descriptive purposes, depending on the normality of distribution (assessed with the Shapiro-Wilk test). Significance level in all the analyses was set at alpha=0.05.
Between-group comparison not accounting for other influences and effect size for difference	We used one-way ANOVA (with Tukey HSD post hoc) and Kruskal-Wallis H (with Dunn post hoc) tests for statistical analysis. To estimate the impact of lifetime BMI trajectory on various health and aging markers, we calculated Cohen's f and epsilon^2 as effect size measures. The effect size (ES) for the ANOVA test was computed as Cohen's f coefficient, which is interpreted as follows: 0.10 = small effect size, 0.25 = moderate effect size, and 0.40 = large effect size [19]. The effect size for the Kruskal-Wallis test was computed as the ε^2 based on the H-statistic: ε^2 [H] = (H - k + 1)/(n - k), where H is the value obtained in the Kruskal-Wallis test; k is the number of groups; n is the total number of observations. The ε^2 estimate assumes values from 0 to 1; multiplied by 100, it indicates the percentage of variance in the dependent variable explained by the independent variable. The interpretation values commonly found in published literature are 0.01- < 0.06 (small effect), 0.06 - < 0.14 (moderate effect), and >= 0.14 (large effect) [57].
Between-group comparison accounting for other influences	The null hypothesis in ANCOVA, which combines ANOVA and regression analysis, states that there is no significant difference in the means of the dependent variable across groups when controlling for a covariate. Essentially, it posits that the independent variable does not affect the outcome, even after accounting for the influence of other predictor variables. The Alternative Hypothesis states a significant difference in the mean of the dependent variable between the groups, after controlling for the covariate. This suggests that the independent variable has a real effect on the outcome, even when the covariate is considered. We performed Tukey's HSD post-hoc test following ANCOVA. Lastly, the effect size (ES) was computed as Cohen's f coefficient.
Cross-sectional within-group comparison	Two-tailed paired Student's t-test compared values of epigenetic and chronological age of the same individual. The Hedges *g* for paired data was computed as a measure of effect size. When using Hedges' g for paired data, the effect size magnitude is interpreted as follows: 0.2 = Small ES; 0.5 = Moderate ES; 0.8 = Large ES; 1.2 = Very Large ES [58].
Longitudinal within-group comparison	ANOVA for repeated measures allowed the comparison of anthropometric and cardiometabolic mean values of correlated samples. In a repeated measures ANOVA, the same participants are measured under multiple conditions or at multiple time points. The factor being manipulated (e.g., different treatments, different time points) is called the within-subjects factor. The null hypothesis (H_0) in a repeated measures ANOVA states that the mean scores are equal across all levels of the within-subjects factor. This means there is no statistically significant difference between the groups. The alternative hypothesis (H_1) is that there is a significant difference in the mean scores across at least two of the levels of the within-subjects

© 2025 Correa-Burrows P et al. *JAMA Network Open*.

	factor. This suggests that the treatment or condition does influence the dependent variable.
Homogeneity in a data series	We conducted the Pettit test for Change-Point Detection to examine whether the DNAmAge series was consistent across the entire sample. This analysis aimed to identify any possible change points within the series that could be attributed to the life-course BMI trajectory. By assessing the homogeneity of the series, we can gain a better understanding of the fundamental patterns and trends of the data. The Pettitt test's underlying hypothesis is that a sequence of observations does or does not exhibit a change of distribution. Specifically, the null hypothesis is that there is no change in distribution (i.e., the data is homogeneous). The alternative hypothesis is that there is a change point, meaning the data series has a shift in its distribution function at some point.
Correlation between epigenetic age and selected anthropometric and body composition markers (eTable 8)	Multiple linear regression models were used to test whether 29-year-olds' sex-adjusted BMI, waist circumference, waist-to-height ratio, and DXA-derived whole body fat mass percentage and truncal fat mass percentage were correlated with epigenetic age as measured by the Horvath Multi-tissue (upper panel) and GrimAge (lower panel) clocks. Results are presented as sex-specific regression coefficients with standard errors reported in parentheses. After regression, our models were validated, including checking the models' 5 assumptions, assessing statistical significance (coefficient significance and Global F test), and evaluating their predictive performance (R-square and adj. R-square). F coefficient and R-sq are reported in eTable 8.

© 2025 Correa-Burrows P et al. *JAMA Network Open*.

eTable 3 Sample description by sex at 29y-assessment of participants (n=205)

	Males (n=105)		Females (n=100)	
	Mean | Median	SD | (25p-75p)	Mean | Median	SD | (25p-75p)
Chronological age (y)	28.9	0.8	28.9	0.6
Body-mass Index	31.5	7.2	32.3	8.6
Waist circumference (cm)	100.3	1.7	92.6	1.9
Waist-to-height ratio	0.58	0.09	0.58	0.11
Systolic BP (mm Hg)	125	(118-132)	117	(108-124)
Diastolic BP (mm Hg)	77	(72-85)	73	(66-79)
VOP (m/s)	5.4	0.3	5.2	0.3
CIMT left (mm)	0.51	0.07	0.48	0.06
CIMT right (mm)	0.48	0.06	0.45	0.05
Fasting glycemia (mg/dl)	94.2	8.9	89.6	9.3
Fasting insulin (μUI/L)	12.8	(9.0-17.7)	11.3	(8.8-18.5)
HOMA-IR	2.75	(2.1-4.2)	2.55	(1.8-4.6)
HOMA-β (%)	137.4	(109-240)	164.8	(127-255)
Triglycerides (mg/dl)	106.1	(75.5-180)	84.7	(61.7-137.5)
HDL-chol (mg/dl)	19.4	(16.4-25)	23.7	(18.6-30.3)
Total cholesterol (mg/dl)	175.5	(146.8-204.6)	166.6	(145.3-199)
MetS Severity Score (SD)	0.85	(.12-1.31)	0.52	(-.03-1.03)
Hamagushi liver score (0-6)	4	(2-5)	3	(2-4)
**BMI category group**				
Group 1 – Always healthy BMI	46	43.3%	43	43.0%
Group 2 – Obesity since adolescence	19	18.1%	24	24.0%
Group 3 – Obesity since childhood	40	38.1%	33	33.0%
**Cardiometabolic-related alterations**				
Insulin resistance	61	58.1%	57	57.0%
Metabolic syndrome	74	70.5%	40	40.0%
NAFLD	54	51.9%	42	42.4%
Low-grade systemic inflammation	44	41.9%	42	42.4%
Type-2 diabetes	1	0.95%	0	0.00%
Metformin	1	0.95%	3	3.00%

Values are expressed as mean and SD, median and (25p-75p) or n and percentage.

© 2025 Correa-Burrows P et al. *JAMA Network Open*.

eTable 4 Prevalence (%) of cardiometabolic comorbidities in the sample by BMI trajectory across the life course

	Group 1 (n=89)	Group 2 (n=43)	Group 3 (n=73)	Pearson's Chi^2 *P* value[a]
Hypertension	26.7	32.6	39.8	.227
Fasting hyperglycemia	9.4	22.5	40.2	.085
Hypertriglyceridemia	14.1	30.0	40.0	.004
Low HDL cholesterol	95.2	100.0	98.6	.246
Metabolic Syndrome	25.0	72.5	68.6	<.001
Insulin resistance	25.0	70.0	77.4	<.001
Fasting hyperinsulinemia	28.1	72.5	74.3	<.001
Fatty liver disease	14.1	63.2	67.1	<.001
Low-grade systemic inflammation	17.2	52.5	63.8	<.001
High cholesterol	23.4	35.0	24.3	.373
Arterial stiffness	0.00	10.5	8.6	.039

Group 1: participants always having a BMI in the healthy range; Group 2: participants with obesity starting in adolescence and remaining obese into adulthood; Group 3: participants who were obese in early childhood and remaining obese into adulthood. Hypertension: SBP>130 mm Hg or DBP>85 mm Hg; fasting hyperglycemia: Gli >100 mg/dl; hypertriglyceridemia; TG>150 mg/dl; low HDL cholesterol: HDL<40 in males and <50 in females; Metabolic Syndrome: AHA/IDF/NBHLI Joint definition; Insulin Resistance: HOMA-IR ≥2.6; fasting hyperinsulinemia: Ins > 12 µUI/L; Fatty liver disease: liver echogenicity (Hamagushi score) ≥4; Low-grade systemic inflammation: hs-CRP >3 mg/L; High cholesterol: >200 mg/dl; Arterial stiffness: Pulse-wave velocity >75th percentile. [a]Pearson's Chi^2 test for independence.

© 2025 Correa-Burrows P et al. *JAMA Network Open*.

eFigure 3 Changes in raw BMI and selected cardiometabolic markers from adolescence to adulthood by BMI trajectory across the life course

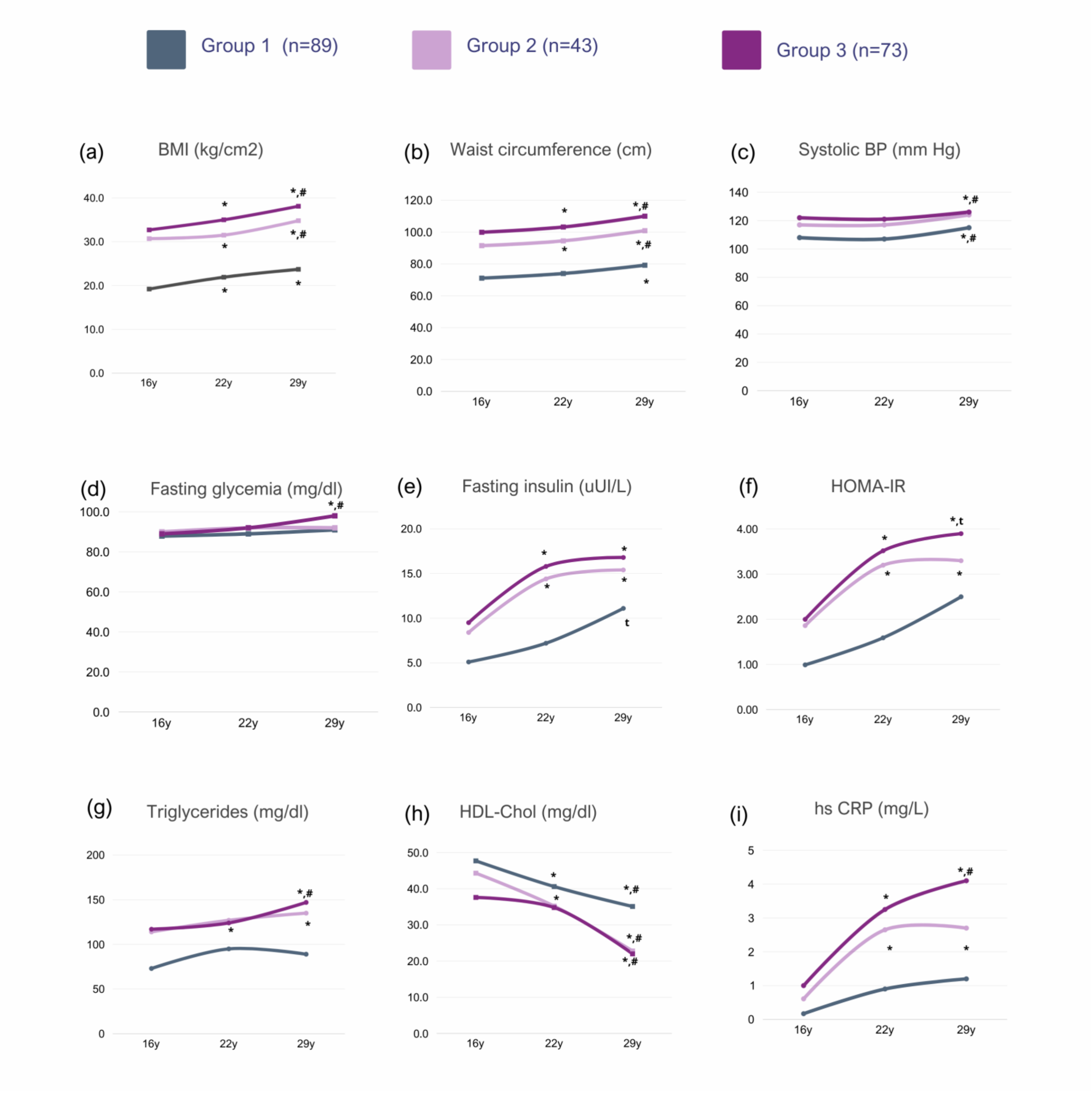

Group 1: participants always having a BMI in the healthy range; Group 2: participants with obesity starting in adolescence and remaining obese into adulthood; Group 3: participants who were obese in early childhood and remaining obese into adulthood. Repeated-measures ANOVA compared trends of raw BMI values and selected cardiometabolic biomarkers assessed at 16, 22, and 29 years. (*) Denotes statistical difference from the given biomarker as measured at 16 years. (#) Denotes statistical difference from the given biomarker as measured at 22 years. (t) Denotes trend toward significance from the given biomarker as measured at 22 years.

© 2025 Correa-Burrows P et al. *JAMA Network Open*.

eAppendix 3 Expanding upon the link between obesity and aging-related epigenetic changes

A shared feature of obesity and aging is the modification of DNA. Environmental factors expose cells to changes in their genomes through epigenetic mechanisms. Over time, these changes accumulate and contribute to the decline observed in older cells. Aging-related epigenetic modifications involve changes in microRNAs, histones, and the methylation of CpG dinucleotides within DNA. Such modifications are associated with greater chromosomal fragility and elevated transcriptional activity noise. The genomic hypomethylation hypothesis of aging suggests that global DNA methylation declines across most, if not all, cells and tissues as both humans and non-human animals age [20-22]. This decline may play a significant role in biological aging, as it disrupts the mechanisms regulating gene expression. The gradual accumulation of genomic alterations (dedifferentiation) impacts cellular homeostasis, instigating a series of harmful reactions that can lead to cell death. Additionally, reduced DNA methylation may influence the risk of developing pathological conditions and diseases such as cancer, atherosclerosis, Alzheimer's disease, and various psychiatric disorders [23].

Numerous studies indicate that obesity leads to notable alterations in gene expression across various tissues and organs [24-26]. Furthermore, an elevated BMI beyond clinically normal levels correlates with changes in the methylation of particular genes [27-28]. In a historical Finnish cohort (Young Finns Study), middle-aged participants with obesity exhibited methylation variations in the DNA of peripheral blood mononuclear cells (PBMCs). Among these individuals, a positive correlation was found between BMI and the epigenetic age of blood cells, implying that higher BMI advances epigenetic aging in this age group [29]. While this correlation was specifically noted in relation to BMI, exploring other body composition metrics and adiposity may provide further insights into how obesity influences epigenetic aging. Horvath et al. highlighted the effect of obesity on epigenetic aging, noting that it speeds up age-related epigenetic alterations in the human liver, leading to an age acceleration of 2.7 years per 10-point rise in BMI [24]. This supports the notion that obesity contributes to faster aging. Moreover, in severely obese Canadian adults (BMI > 40 kg/m^2), an increase in epigenetic age acceleration in visceral adipose tissue was observed alongside rising BMI, indicating that obesity is linked to biological age acceleration in metabolically active tissues [25]. Lastly, a meta-analysis by Oblak et al. found a correlation between BMI and elevated epigenetic age across the Horvath, Levine, and GrimAge clocks [30]. Notably, DNA methylation could predict up to 20% of the variation in BMI [30]. The Metabolic Syndrome (MetS), closely linked to BMI, is a cluster of metabolic issues that significantly increases the risk of type 2 diabetes mellitus (T2DM) and cardiovascular diseases. In the American participants of the CARDIA (Coronary Artery Risk Development in Young Adults Study), a longitudinal study focused on cardiovascular disease development and its risk factors from adolescence, Nannini et al. identified a highly significant impact of MetS on both intrinsic (IEA_{accel}) and extrinsic (EEA_{accel}) epigenetic age acceleration [31]. Their cross-sectional analysis demonstrated that a higher MetS severity score correlated positively with IEA_{accel} at ages 15 and 20, and with EEA_{accel} at age 20. Intrinsic epigenetic age pertains to cellular changes during biological aging, while extrinsic epigenetic age indicates the aging of the immune system. All these studies indicate a possible link between obesity and age-related changes in epigenetic markers. Nonetheless, they were carried out solely in Northern Hemisphere populations, potentially overlooking the environmental factors that might impact the Latin American population, and specifically the Chilean population.

On a mechanistic level, it is worth recalling that aging-related hypomethylation is recognized as a primary hallmark of aging. Over time, damage to the epigenome builds up, leading to the activation of additional aging hallmarks. Obesity can accelerate the progression to the subsequent stage of aging hallmarks, referred to as antagonistic hallmarks [32]. For instance, obesity may intensify trophic signaling and anabolic processes triggered by nutrient sensing [33,34]. These signals have beneficial actions in youth but are largely pro-ageing later on. Thus, the nutrient-sensing network contributes to growth and development until young adulthood but plays a detrimental role beyond this stage [35]. Since our participants are transitioning from early to middle adulthood, and those exposed to obesity show a mean gap of >15% between chronological and epigenetic aging, nutrient sensing signaling may no longer work as a process that benefits an organism's development, but rather as a pro-aging factor. Likewise, obesity-induced epigenetic aging may promote cell senescence. Cellular senescence refers to a loss of proliferative capacity, later linked to telomere shortening. Senescence in adult tissues can be triggered by factors beyond replicative exhaustion, including a persistent DNA damage response induced by various internal and external factors, including obesity [36,37]. White adipose tissue (WAT) cells are highly susceptible to senescence with aging but also with obesity, regardless of chronological age. WAT senescence is associated with inappropriate expansion (hypertrophy) of adipocytes, insulin resistance, and dyslipidemia, which further exacerbates this pro-aging phenotype. When cells enter senescence, they not only cease to proliferate but also increase the secretion of inflammatory molecules known as the Senescence-Associated Secretory Phenotype (SASP). Both senescence and SASP are linked to various age-related conditions diseases [38].

© 2025 Correa-Burrows P et al. *JAMA Network Open*.

eTable5 Aging-related cytokines, adipokines, myokines, and growth factors in participants by BMI trajectory (n=205)

	Group 1 (n=89)	Group 2 (n=43)	Group 3 (n=73)	Between-group Differences[b]
hs CRP (mg/l)[ac]	1.69 (2.1)	3.67 (2.8)	4.24 (2.4)	ABB
IL-2	1.48 (1.21-2.17)	1.67 (1.40-2.00)	1.72 (1.56-2.33)	ABB
IL-6	1.76 (1.54-2.54)	2.58 (1.87-3.73)	2.35 (1.92-4.13)	ABB
IL-10	7.87 (6.58-12.2)	9.07 (7.47-12.2)	9.77 (8.01-13.8)	ABB
TNF α	11.2 (9.8-13.3)	11.5 (10.3-13.8)	11.8 (10.3-16.1)	*NS*
FGF-21	193.6 (116.8-299.2)	288.0 (180.8-358.4)	292.8 (203.2-425.6)	ABB
GDF-11	16.4 (15.2-17.6)	17.2 (15.2-18.4)	17.2 (15.6-18.4)	*NS*
GDF-15	2,879.2 (2,081.6-3612.8)	3,009.2 (2,420.8-3,555.2)	3,299.2 (2,630.4-3,828.8)	AAC
IGF-1	45,000 (35,712-53,028)	36,936 (29,304-44,784)	30,204 (23,580-37,728)	ABC
IGF-2	376,452 (333,972-407,160)	301,140 (252000-333792)	270,108 (228,420-344,988)	ABB
Leptin - males	7,624 (4,508-17,216)	12,756 (10,976-37,512)	18,986 (12,396-33,256)	ABB
Leptin - females	19,606 (14,436-32,774)	55,498 (22,582-75,680)	57,876 (31,536-94,452)	ABB
Apelin	262.2 (228.6-410.4)	295.2 (249.7-343.2)	274.5 (236.0-315.7)	ABB
Myostatin	1,113.5 (1,022-1,348.5)	1,282 (1,123.5-1,558)	1,232.5 (1,068.5-1,489)	ABB
Irisin (pg/ml)	2,218.5 (1,991- 2,511)	2,348.5 (2,166.5-3,112)	2,293.5 (2,074-2,726.5)	ABB
Oncostatin	5.1 (4.4-6.4)	5.6 (4.8-6.6)	5.4 (4.5-6.8)	ABB

Values are expressed as median (25p-75p). [a] Values are mean (SD). Group 1: participants always having a BMI in the healthy range; Group 2: participants with obesity starting in adolescence and remaining obese into adulthood; Group 3: participants who were obese in early childhood and remained obese into adulthood. [b] Kruskal-Wallis H test with Dunn post hoc adjustment, except otherwise indicated. [c] ANOVA with Tukey post hoc adjustment for mean comparison. Post-hoc analysis for between-group differences (Dunn and Tukey post hoc): A=Group 1; B=Group 2; C=Group 3. Different letters indicate significant statistical differences. The same letter denotes means that do not differ. For instance, ABB indicates that Group 1 has values significantly different from those of Group 2 and Group 3, while the mean values in Group 2 and Group 3 do not differ.

© 2025 Correa-Burrows P et al. *JAMA Network Open*.

eAppendix 4 Expanding upon obesity-related cytokines, adipokines, myokines and growth factors profile as molecular aging signatures

Obesity is characterized by low-grade inflammation, which leads to the release of pro-inflammatory cytokines, human growth factors (GF), abnormal myokines, and other adipokines from adipose tissue. Chronic low-grade inflammation is also a common feature of aging, increasing the risk of premature morbidity and mortality [39-40]. We determined a selection of such biomarkers in ObAGE participants and compared their expression levels between different BMI trajectory groups (see Table 4 for log-transformed variables and eTable 6 for variables expressed in their actual concentration units). In our sample, obesity persisting since childhood or adolescence into adulthood significantly impacted proteins such as hsCRP, IL6, and leptin and had a moderate-to-strong effect on IL2 and IL10, all well-known biomarkers of systemic inflammation. Conversely, it did not affect the expression of TNFα and GDF-11. GDF-11 typically decreases with age, but it was also found to remain unchanged in individuals with obesity and T2D [41]. TNFα plays a crucial role in the link between obesity and diabetes. It may be necessary to have severe insulin resistance or even T2D for TNFα to be significantly upregulated in individuals with obesity, especially if they are young [42]. Systemic inflammation is a newly recognized hallmark of aging, stemming from genomic instability, epigenetic dysregulation, impaired proteostasis, insufficient autophagy, or the buildup of senescent cells [32]. Inflammation, in turn, paves the way for other signs of aging. For instance, it plays a significant role in intercellular signaling and the immune system's ability to combat age-related damage. In our sample, participants with obesity since childhood or adolescence presented dysregulated values of several myokines (Apelin, Irisin, Myostatin, Oncostatin, Musclin, and Osteonectin), a group of proteins able to mediate muscle–organ crosstalk to the brain, adipose tissue, bone, liver, gut, pancreas, vascular bed, and skin [43,44]. Obesity and aging are associated with dysregulated myokine secretion and signaling, contributing to skeletal muscle loss and metabolic disturbances. Thus, it should not be surprising that these markers of intercellular communication are increased in obese subjects compared to normal weight controls, particularly apelin and irisin, which tend to decline in the aging organism. In a developing organism, such as our participants, the increase in these myokines may be a response to metabolic dysfunction induced by obesity. This upregulation could be a reaction to improve insulin sensitivity in obese individuals, or it could be the consequence of reduced sensitivity to its effects, as seen with insulin and leptin in obesity [45,46]. At the systemic level, inflammation is associated with several health issues, including endothelial dysfunction, insulin resistance, hepatic steatosis, and metabolic syndrome. In our study, individuals with obesity since childhood or adolescence exhibited markers of these alterations, such as elevated values of systolic blood pressure, diastolic blood pressure, and pulse-wave velocity; insulin, HOMA-IR, and HOMA-β; Hamagushi liver score; and MetS severity score. All these changes are the precursor to age-related diseases; thus, it is reasonable to assume that just as older individuals exhibit frailty as a sign of aging, younger individuals may exhibit dysfunction and cardiometabolic damage as initial signs of aging. Also noteworthy is the effect size for the difference in two components of the insulin-like growth factor family, IGF1 and IGF2, and the direction of their relationship with the BMI trajectory. Based on Cohen's f values, IGF2 was the non-epigenetic biomarker of aging most affected by long-term obesity or obesity persisting since childhood or adolescence. Participants suffering from this condition from early life stages show decreased values of IGF2 in adulthood. Decreased expression of this growth factor has been linked to the aging of various organs and primordial germ cells. It is postulated that reduced IGF2 compromises the functionality of the mitochondria [47,48]. IGF1 was also found to be reduced in participants with OCA. Earlier studies suggested a link between high IGF-1 levels and negative outcomes related to mortality and age-related health issues [49]. However, subsequent research has shown that the advantages of lower IGF-1 are more visible in older individuals. In their analysis of data from the UK Biobank from individuals in various age groups, Zhang et al. discovered that IGF1 is a non-linear risk indicator that interacts with age to alter the risk for various health outcomes. Specifically, elevated IGF-1 is mainly linked to reduced disease risk in younger individuals [50]. Conversely, it is associated with higher illness and death rates in older individuals [51]. Therefore, lower IGF1 levels in participants with OCA in our sample might suggest a higher risk of health issues consistent with an altered cardiometabolic profile. According to some reports, a possible explanation is that IGF-1 levels decrease with age, so higher IGF1 in young adulthood can be considered a youth biomarker [51,53,54]. Diminished IGF1 levels in the elderly lead to loss of resiliency, while IGF1 is crucial for normal development in younger individuals, as the focus is on growth and expansion during youth rather than preservation [55].

© 2025 Correa-Burrows P et al. *JAMA Network Open*.

eAppendix 5 Expanding upon the clinical implications of our findings

One of the key clinical implications of our findings is the ability to detect early physiological disturbances through a laboratory-based biomarker, even in the absence of abnormal clinical parameters. In our cohort, young adults with obesity had normal levels of blood pressure, fasting glucose, and total cholesterol (Table 1)—values that could lead clinicians to underestimate the health risks due to the patient's age. However, their accelerated epigenetic aging unmasks an underlying physiological impact, highlighting the limitations of conventional biomarkers in this population. Although our study did not directly assess long-term outcomes—given that participants were young and had not yet developed overt chronic diseases such as myocardial infarction, heart failure, or chronic kidney disease—these findings suggest that epigenetic clocks may serve as early indicators of future cardiometabolic risk. Longitudinal studies will be essential to validate this hypothesis. Importantly, whether lifestyle or pharmacologic interventions for obesity can reverse or mitigate the observed epigenetic alterations remains an open question with major implications for translational aging research. Clarifying this point in future trials may inform novel risk stratification strategies and provide mechanistic insights into the biology of resilience in young individuals with obesity. Also, the presence of molecular aging markers coupled with a dysfunctional cardiometabolic profile in developing organisms strongly supports the concept that health encompasses a set of organizational and dynamic characteristics that maintain physiology rather than simply denoting the absence of pathology [56]. For instance, in our sample, only one subject had T2D diagnosis, while 70% showed impaired insulin sensitivity. Only three of those with impaired insulin sensitivity were using metformin. Based on this example, our cohort could be considered healthy if health is defined as the absence of disease. However, delving into the cell and molecular levels reveals a markedly different reality, where homeostatic maintenance seems to exist in a weak balance. Future studies should explore how long this balance can be sustained and what preventative or therapeutic actions can delay the transition to age-related multimorbidity. It is crucial to investigate these questions as disease-free young adults, who will reach the prime of middle adulthood within the next 10-15 years, are already displaying clear signs of age-related physiological disruption.

© 2025 Correa-Burrows P et al. *JAMA Network Open*.

eTable6 Epigenetic aging-related profile in participants with long-term obesity and free of cardiometabolic comorbidities

	Overall sample (n=20)	Males (n=8)	Females (n=12)
Chronological age (y)	28.9 (0.9)	28.9 (0.8)	28.6 (0.7)
DNAm age (y)	34.0 (3.6)	33.4 (2.6)	34.5 (4.2)
DNAm age Horvath acceleration (y)	5.32 (3.4)	4.48 (2.5)	5.89 (3.9)
DNAm age Horvath acceleration (%)	18.5 (11.8)	15.6 (8.9)	20.5 (13.3)

Values are expressed as mean (SD). Free of cardiometabolic comorbidities means having neither Metabolic Syndrome nor fatty liver disease.

© 2025 Correa-Burrows P et al. *JAMA Network Open*.

eTable7 Correlation between sex-adjusted anthropometric and DXA-derived body composition markers with epigenetic age in participants (n=205)

	Epigenetic age (dependent variable) as measured by the DNAm age (y)				
**Predictors**	**BMI (kg/m2)**	**WC (cm)**	**WtHt**	**Fat mass (%)**	**Trunkal fat mass (%)**
Intercept	22.2*** (1.19)	19.5*** (1.7)	18.9*** (1.7)	21.2*** (1.5)	22.9*** (1.41)
Predictor#Males	0.32*** (.04)	0.13*** (.02)	.23*** (.03)	0.31*** (.04)	0.30*** (.05)
Predictor#Females	0.25*** (.04)	0.11*** (.02)	.20*** (.03)	0.22*** (.03)	0.24*** (.04)
F global test (*p*)	35.5 (<.001)	28.6 (<.001)	32.7 (<.001)	28.7 (<.001)	22.7 (<.001)
R-sq	0.29	0.25	0.28	0.26	0.21

	Epigenetic age (dependent variable) as measured by the DNAm GrimAge (y)				
**Predictors**	**BMI (kg/m2)**	**WC (cm)**	**WtHt**	**Fat mass (%)**	**Trunkal fat mass (%)**
Intercept	21.0*** (1.3)	17.7*** (1.7)	17.3*** (1.6)	20.0*** (1.6)	21.1*** (1.5)
Predictor#Males	0.29*** (.01)	0.12*** (.02)	0.21*** (.03)	0.27*** (.05)	0.29*** (.05)
Predictor#Females	0.25*** (.01)	0.13*** (.02)	0.20*** (.03)	0.19*** (.05)	0.24*** (.05)
F global test (*p*)	25.3 (<.001)	24.7 (<.001)	26.9 (<.001)	17.3 (<.001)	17.7 (<.001)
R-sq	0.24	0.22	0.23	0.18	0.18

Multiple linear regression models were used to test whether 29-year-olds' sex-adjusted BMI, waist circumference, waist-to-height ratio, and DXA-derived whole body fat mass percentage and truncal fat mass percentage were correlated with epigenetic age as measured by the Horvath Multi-tissue (upper panel) and GrimAge (lower panel) clocks. Standard errors are reported in parentheses. *** indicates significance at the 99.9% level.

The results showed that BMI, WC, WtHtR, Fat Mass (%) and truncal fat mass could predict 25% to 29% of DNAmAge variation in adulthood. Meanwhile, they could predict 18% to 24% of GrimAge variation. Interestingly, BMI exhibited the highest predictive ability among all anthropometric markers when predicting epigenetic age, regardless of the clock used for its estimation. Despite BMI being recognized to have significant limitations as a predictor of cardiometabolic risk due to its inability to capture fat quantity and distribution, this finding could have important epidemiological implications. Other influences, such as biological, behavioral, and socioeconomic factors, should be considered in future studies to confirm this finding. Also of note was that the sex-specific regression coefficients were significant for all markers in all models.

© 2025 Correa-Burrows P et al. *JAMA Network Open*.

eREFERENCES

1. Lozoff B, De Andraca I, Castillo M, Smith JB, Walter T, Pino P. Behavioral and developmental effects of preventing iron-deficiency anemia in healthy full-term infants. Pediatrics. 2003 Oct;112(4):846-54. PMID: 14523176.
2. Albala C, Vio F, Kain J, Uauy R. Nutrition transition in Chile: determinants and consequences. Public Health Nutr. 2002 Feb;5(1A):123-8. doi: 10.1079/PHN2001283.
3. Albala C, Vio F, Kain J, Uauy R. Nutrition transition in Latin America: the case of Chile. Nutr Rev. 2001 Jun;59(6):170-6. doi: 10.1111/j.1753-4887.2001.tb07008.x.
4. Sun W, Joffe MM, Chen J, Brunelli SM. Design and analysis of multiple events case-control studies. Biometrics. 2010;66(4):1220-1229. doi:10.1111/j.1541-0420.2009.01369.x
5. WHO Multicentre Growth Reference Study Group. WHO Child Growth Standards based on length/height, weight and age. Acta Paediatr Suppl. 2006;450:76-85. doi:10.1111/j.1651-2227.2006.tb02378.x
6. de Onis M, Onyango AW, Borghi E, Siyam A, Nishida C, Siekmann J. Development of a WHO growth reference for school-aged children and adolescents. *Bull World Health Organ*. 2007;85(9):660-667. doi:10.2471/blt.07.043497
7. Emery W, Thompson R. Statistical Methods and Error Handling. in: Data Analysis Methods in Physical Oceanography. 193–304 (Elsevier, 2001). DOI: 10.1016/B978-044450756-3/50004-6.
8. Pollock D. Smoothing with Cubic Splines. in: Handbook of Time Series Analysis, Signal Processing, and Dynamics. 293–322 (Academic Press, 1999). DOI: 10.1016/B978-012560990-6/50013-0.
9. Correa-Burrows P, Rogan J, Blanco E, et al. Resolving early obesity leads to a cardiometabolic profile within normal ranges at 23 years old in a two-decade prospective follow-up study. Sci Rep. 2021;11(1):18927. Published 2021 Sep 23. doi:10.1038/s41598-021-97683-9.
10. Whelton, P., Carey, R., Aronow, F., et al. Guideline for the prevention, detection, evaluation, and management of high blood pressure in adults: a report of the American College of Cardiology/American Heart Association Task Force on Clinical Practice Guidelines *Hypertension* 71, e13–e115 (2018). DOI: 10.1161/HYP.0000000000000065
11. Bia, D. & Zócalo, Y. Physiological Age- and Sex-Related Profiles for Local (Aortic) and Regional (Carotid-Femoral, Carotid-Radial) Pulse Wave Velocity and Center-to-Periphery Stiffness Gradient, with and without Blood Pressure Adjustments: Reference Intervals and Agreement between Methods in Healthy Subjects (3-84 Years). *J Cardiovasc Dev Dis* 3 (2021). DOI:10.3390/jcdd8010003
12. Roberts WL; CDC; AHA. CDC/AHA Workshop on Markers of Inflammation and Cardiovascular Disease: Application to Clinical and Public Health Practice: A background paper: laboratory tests available to assess inflammation--performance and standardization. *Circulation* 110, e572-e576 (2004). DOI:10.1161/01.CIR.0000148986.52696.07
13. Burrows, R., Correa, P., Reyes. M., Blanco, E., Albala, C., Gahagan, S. Healthy Chilean Adolescents with HOMA-IR ≥ 2.6 Have Increased Cardiometabolic Risk: Association with Genetic, Biological, and Environmental Factors. *J Diabetes Res* 2015, 783296 (2015). DOI:10.1155/2015/783296
14. Alberti KG, Eckel RH, Grundy SM, et al. Harmonizing the metabolic syndrome: a joint interim statement of the International Diabetes Federation Task Force on Epidemiology and Prevention; National Heart, Lung, and Blood Institute; American Heart Association; World Heart Federation; International Atherosclerosis Society; and International Association for the Study of Obesity. *Circulation*. 120, 1640-1645 (2009). DOI:10.1161/CIRCULATIONAHA.109.192644
15. DeBoer, M. & Gurka, M. Clinical utility of metabolic syndrome severity scores: considerations for practitioners. *Diabetes Metab Syndr Obes* 10, 65-72 (2017). DOI: 10.2147/DMSO.S101624.
16. Hamaguchi, M., Kojima, T., Itoh, Y., et al. The severity of ultrasonographic findings in nonalcoholic fatty liver disease reflects the metabolic syndrome and visceral fat accumulation. *Am J Gastroenterol* 102, 2708-15 (2007). doi: 10.1111/j.1572-0241.2007.01526.x.

© 2025 Correa-Burrows P et al. *JAMA Network Open*.

17. Ibacahe, C., Correa, P., Burrows, R., et al. Accuracy of a Semi-Quantitative Ultrasound Method to Determine Liver Fat Infiltration in Early Adulthood. *Diagnostics* 10, 431 (2020). DOI: 10.3390/diagnostics10060431.

18. Stein, J., Korcarz, C., Hurst, R., et al. Use of carotid ultrasound to identify subclinical vascular disease and evaluate cardiovascular disease risk: a consensus statement from the American Society of Echocardiography Carotid Intima-Media Thickness Task Force. *J Am Soc Echocardiogr* 21, 93-190 (2008). DOI:10.1016/j.echo.2007.11.011.

19. Steiger JH. Beyond the F test: Effect size confidence intervals and tests of close fit in the analysis of variance and contrast analysis. Psychol Methods. 2004 Jun;9(2):164-82. doi: 10.1037/1082-989X.9.2.164.

20. Unnikrishnan A, Hadad N, Masser DR, Jackson J, Freeman WM, Richardson A. Revisiting the genomic hypomethylation hypothesis of aging. Ann N Y Acad Sci. 2018 Apr;1418(1):69-79. doi: 10.1111/nyas.13533.

21. Pogribny IP, Beland FA. DNA hypomethylation in the origin and pathogenesis of human diseases. Cell Mol Life Sci. 2009 Jul;66(14):2249-61. doi: 10.1007/s00018-009-0015-5.

22. Heyn H, Li N, Ferreira HJ, Moran S, Pisano DG, Gomez A, Diez J, Sanchez-Mut JV, Setien F, Carmona FJ, Puca AA, Sayols S, Pujana MA, Serra-Musach J, Iglesias-Platas I, Formiga F, Fernandez AF, Fraga MF, Heath SC, Valencia A, Gut IG, Wang J, Esteller M. Distinct DNA methylomes of newborns and centenarians. Proc Natl Acad Sci U S A. 2012 Jun 26;109(26):10522-7. doi: 10.1073/pnas.1120658109

23. Vanyushin BF, Nemirovsky LE, Klimenko VV, Vasiliev VK, Belozersky AN. The 5-methylcytosine in DNA of rats. Tissue and age specificity and the changes induced by hydrocortisone and other agents. Gerontologia. 1973;19(3):138-52. PMID: 4763637.

24. Horvath S, Erhart W, Brosch M, et al. Obesity accelerates epigenetic aging of human liver. Proc Natl Acad Sci U S A. 2014 Oct 28;111(43):15538-43. doi: 10.1073/pnas.1412759111.

25. De Toro-Martín J, Guénard F, Tchernof A, et al. Body mass index is associated with epigenetic age acceleration in the visceral adipose tissue of subjects with severe obesity. Clin Epigenetics. 2019 Dec 2;11(1):172. doi: 10.1186/s13148-019-0754-6.

26. Wang X, Zhu H, Snieder H, Su S, Munn D, Harshfield G, et al. Obesity related methylation changes in DNA of peripheral blood leukocytes. BMC Med. (2010) 8:87. doi: 10.1186/1741-7015-8-87

27. Kaushik P, Anderson JT. Obesity: epigenetic aspects. Biomol Concepts. 2016 Jun 1;7(3):145-55. doi: 10.1515/bmc-2016-0010.

28. Richmond RC, Sharp GC, Ward ME, Fraser A, Lyttleton O, McArdle WL, Ring SM, Gaunt TR, Lawlor DA, Davey Smith G, Relton CL. DNA Methylation and BMI: Investigating Identified Methylation Sites at HIF3A in a Causal Framework. Diabetes. 2016 May;65(5):1231-44. doi: 10.2337/db15-0996.

29. Nevalainen T, Kananen L, Marttila S, Jylhävä J, Mononen N, Kähönen M, Raitakari OT, Hervonen A, Jylhä M, Lehtimäki T, Hurme M. Obesity accelerates epigenetic aging in middle-aged but not in elderly individuals. Clin Epigenetics. 2017 Feb 14;9:20. doi: 10.1186/s13148-016-0301-7.

30. Oblak L, van der Zaag J, Higgins-Chen AT, Levine ME, Boks MP. A systematic review of biological, social and environmental factors associated with epigenetic clock acceleration. Ageing Res Rev. 2021 Aug;69:101348. doi: 10.1016/j.arr.2021.101348.

31. Nannini DR, Joyce BT, Zheng Y, et al. Epigenetic age acceleration and metabolic syndrome in the coronary artery risk development in young adults study. Clin Epigenetics. 2019 Nov 15;11(1):160. doi: 10.1186/s13148-019-0767-1.

32. López-Otín C, Blasco MA, Partridge L, Serrano M, Kroemer G. Hallmarks of aging: An expanding universe. Cell. 2023 Jan 19;186(2):243-278. doi: 10.1016/j.cell.2022.11.001

33. Oldham S. Obesity and nutrient sensing TOR pathway in flies and vertebrates: Functional conservation of genetic mechanisms. Trends Endocrinol Metab. 2011 Feb;22(2):45-52. doi: 10.1016/j.tem.2010.11.002.

34. Obici S, Rossetti L. Minireview: nutrient sensing and the regulation of insulin action and energy balance. Endocrinology. 2003 Dec;144(12):5172-8. doi: 10.1210/en.2003-0999. Epub 2003 Sep 11. PMID: 12970158

© 2025 Correa-Burrows P et al. *JAMA Network Open*.

35. Sung Y, Yu YC, Han JM. Nutrient sensors and their crosstalk. Exp Mol Med. 2023 Jun;55(6):1076-1089. doi: 10.1038/s12276-023-01006-z.

36. Narasimhan A, Flores RR, Camell CD, Bernlohr DA, Robbins PD, Niedernhofer LJ. Cellular Senescence in Obesity and Associated Complications: a New Therapeutic Target. Curr Diab Rep. 2022 Nov;22(11):537-548. doi: 10.1007/s11892-022-01493-w.

37. Smith U, Li Q, Rydén M, Spalding KL. Cellular senescence and its role in white adipose tissue. Int J Obes (Lond). 2021 May;45(5):934-943. doi: 10.1038/s41366-021-00757-x.

38. Dasgupta N, Arnold R, Equey A, Gandhi A, Adams PD. The role of the dynamic epigenetic landscape in senescence: orchestrating SASP expression. NPJ Aging. 2024 Oct 24;10(1):48. doi: 10.1038/s41514-024-00172-2.

39. Franceschi C, Garagnani P, Parini P, Giuliani C, Santoro A. Inflammaging: a new immune-metabolic viewpoint for age-related diseases. Nat Rev Endocrinol. 2018;14(10):576-590. doi:10.1038/s41574-018-0059-4

40. Fulop T, Witkowski JM, Olivieri F, Larbi A. The integration of inflammaging in age-related diseases. *Semin Immunol*. 2018;40:17-35. doi:10.1016/j.smim.2018.09.003

41. Añón-Hidalgo, J., Catalán, V., Rodríguez, A., et al. Circulating GDF11 levels are decreased with age but are unchanged with obesity and type 2 diabetes. *Aging* 11, 1733-1744 (2019). DOI: 10.18632/aging.101865.

42. Alzamil H. Elevated Serum TNF-α Is Related to Obesity in Type 2 Diabetes Mellitus and Is Associated with Glycemic Control and Insulin Resistance. J Obes. 2020 Jan 30;2020:5076858. doi: 10.1155/2020/5076858.

43. Pedersen, L. & Hojman, P. Muscle-to-organ cross-talk mediated by myokines. *Adipocyte* 1, 64-167 (2012). DOI: 10.4161/adip.20344.

44. Severinsen, M. & Pedersen, B. Muscle-Organ Crosstalk: The Emerging Roles of Myokines. *Endocr Rev* 41, 594–609 (2020). DOI: 10.1210/endrev/bnaa016.

45. Jia, J., Yu, F., Wei, W., Yang, P., Zhang, R., Sheng, Y. & Shi, Y. Relationship between circulating irisin levels and overweight/obesity: A meta-analysis. *World J Clin Cases 7*, 1444-1455 (2019). DOI: 10.12998/wjcc.v7.i12.1444.

46. Li, C., Cheng, H., Adhikari, B., et al. The Role of Apelin-APJ System in Diabetes and Obesity. *Front Endocrinol* 13, 820002 (2022). DOI: 10.3389/fendo.2022.820002.

47. Muhammad T, Wan Y, Sha Q, Wang J, Huang T, Cao Y, Li M, Yu X, Yin Y, Chan WY, Chen ZJ, You L, Lu G, Liu H. IGF2 improves the developmental competency and meiotic structure of oocytes from aged mice. Aging (Albany NY). 2020 Dec 9;13(2):2118-2134. doi: 10.18632/aging.202214.

48. Zhou X, Tan B, Gui W, Zhou C, Zhao H, Lin X, Li H. IGF2 deficiency promotes liver aging through mitochondrial dysfunction and upregulated CEBPB signaling in D-galactose-induced aging mice. Mol Med. 2023 Nov 28;29(1):161. doi: 10.1186/s10020-023-00752-0.

49. Milman S, Atzmon G, Huffman DM, Wan J, Crandall JP, Cohen P, Barzilai N. Low insulin-like growth factor-1 level predicts survival in humans with exceptional longevity. Aging Cell. 2014 Aug;13(4):769-71. doi: 10.1111/acel.12213.

50. Zhang WB, Aleksic S, Gao T, Weiss EF, Demetriou E, Verghese J, Holtzer R, Barzilai N, Milman S. Insulin-like Growth Factor-1 and IGF Binding Proteins Predict All-Cause Mortality and Morbidity in Older Adults. Cells. 2020 Jun 1;9(6):1368. doi: 10.3390/cells9061368.

51. Zhang WB, Milman S. Looking at IGF-1 through the hourglass. Aging (Albany NY). 2022 Aug 25;14(16):6379-6380. doi: 10.18632/aging.204257.

52. Zhang WB, Ye K, Barzilai N, Milman S. The antagonistic pleiotropy of insulin-like growth factor 1. Aging Cell. 2021 Sep;20(9):e13443. doi: 10.1111/acel.13443.

53. Gubbi S, Quipildor GF, Barzilai N, Huffman DM, Milman S. 40 YEARS of IGF1: IGF1: the Jekyll and Hyde of the aging brain. J Mol Endocrinol. 2018 Jul;61(1):T171-T185. doi: 10.1530/JME-18-0093.

© 2025 Correa-Burrows P et al. *JAMA Network Open*.

54. Lakens D. Calculating and reporting effect sizes to facilitate cumulative science: a practical primer for t-tests and ANOVAs. *Front Psychol*. 2013;4:863. Published 2013 Nov 26. doi:10.3389/fpsyg.2013.00863

55. Selya AS, Rose JS, Dierker LC, Hedeker D, Mermelstein RJ. A Practical Guide to Calculating Cohen's f(2), a Measure of Local Effect Size, from PROC MIXED. Front Psychol. 2012 Apr 17;3:111. doi: 10.3389/fpsyg.2012.00111. PMID: 22529829; PMCID: PMC3328081.

56. López-Otín C, Kroemer G. Hallmarks of Health. Cell. 2021 Jan 7;184(1):33-63. doi: 10.1016/j.cell.2020.11.034. Epub 2020 Dec 18. Erratum in: Cell. 2021 Apr 1;184(7):1929-1939. doi: 10.1016/j.cell.2021.03.033.

57. Kelley T. An Unbiased Correlation Ratio Measure. Proceedings of the National Academy of Sciences of the United States of America, 1935, 21(9): 554–559.

58. Gibbons, R., Hedeker, D. & Davis, J. Estimation of effect size from a series of experiments involving paired comparisons. J Educ Stat 18, 271–279 (1993). DOI: 10.3102/10769986018003271

© 2025 Correa-Burrows P et al. *JAMA Network Open*.
