# Supplementary material for: Long-Term Obesity and Biological Aging in Young Adults
Source: JAMA Netw Open. 2025 Jul 11;8(7):e2520011. doi: 10.1001/jamanetworkopen.2025.20011 (PMC12254895; doi:10.1001/jamanetworkopen.2025.20011)
Supplement: Supplement 2. — Data Sharing Statement [file jamanetwopen-e2520011-s002.pdf]

Data Sharing Statement

Correa-Burrows. Long-Term Obesity and Biological Aging in Young Adults. *JAMA Netw Open*. Published July 11, 2025. doi:10.1001/jamanetworkopen.2025.20011

Data
Data available: Yes
Data types: Deidentified participant data
How to access data: The data will be available on request from the corresponding authors.
When available: With publication

Supporting Documents
Document types: None

Additional Information
Who can access the data: Data will be available only to researchers whose proposed use of the data had been approved by the corresponding authors.
Types of analyses: Data will be available only for scientific research purposes.
Mechanisms of data availability: Data will be made available with investigator support, after approval of a proposal AND with a signed data access agreement
